# Discovery of a dual protease mechanism that promotes DNA damage checkpoint recovery

**DOI:** 10.1371/journal.pgen.1007512

**Published:** 2018-07-06

**Authors:** Peter E. Burby, Zackary W. Simmons, Jeremy W. Schroeder, Lyle A. Simmons

**Affiliations:** Department of Molecular, Cellular, and Developmental Biology, University of Michigan, Ann Arbor, MI, United States of America; Université Paris Descartes, INSERM U1001, FRANCE

## Abstract

The DNA damage response is a signaling pathway found throughout biology. In many bacteria the DNA damage checkpoint is enforced by inducing expression of a small, membrane bound inhibitor that delays cell division providing time to repair damaged chromosomes. How cells promote checkpoint recovery after sensing successful repair is unknown. By using a high-throughput, forward genetic screen, we identified two unrelated proteases, YlbL and CtpA, that promote DNA damage checkpoint recovery in *Bacillus subtilis*. Deletion of both proteases leads to accumulation of the checkpoint protein YneA. We show that DNA damage sensitivity and increased cell elongation in protease mutants depends on *yneA*. Further, expression of YneA in protease mutants was sufficient to inhibit cell proliferation. Finally, we show that both proteases interact with YneA and that one of the two proteases, CtpA, directly cleaves YneA *in vitro*. With these results, we report the mechanism for DNA damage checkpoint recovery in bacteria that use membrane bound cell division inhibitors.

## Author summary

Prokaryotes and eukaryotes coordinate cell division to genome integrity using DNA damage checkpoints. Many bacteria express a small, membrane binding protein to slow cell division when obstacles to DNA replication are encountered. Cell division inhibitors of this class have been identified in several bacterial species, yet the mechanism used to alleviate inhibition has remained unknown. Using forward genetics, we identified two unstudied genes, coding for the proteases YlbL and CtpA, that when deleted result in sensitivity to drugs that directly damage DNA. We show that sensitivity to DNA damage in protease mutants is a result of accumulation of the cell division inhibitor. Further, we show that YlbL and CtpA are responsible for degrading the cell division inhibitor allowing for cell division to resume. Importantly, these two proteases are not homologs, demonstrating a striking example of a bacterium using non-homologous enzymes to degrade a single substrate. Our investigation uncovers the previously unknown mechanism used to remove a cell division inhibitor, while also illuminating a potential strategy that bacteria can use to regulate signaling pathways. The use of multiple, unrelated proteins to perform a single function may represent a strategy employed throughout biological systems.

## Introduction

The DNA damage response (DDR, SOS response in bacteria) is an important pathway for maintaining genome integrity in all domains of life. Misregulation of the DDR in humans can result in various disease conditions [1, 2], and in bacteria the SOS response has been found to be important for survival under many stressors [3–5]. The DNA damage response in all organisms results in three principle outcomes: a transcriptional response, which can vary depending on the type of DNA damage incurred, DNA repair, and activation of a DNA damage checkpoint [6–9]. In eukaryotes, the G1/S and G2/M checkpoints are established by checkpoint kinases, which transduce the signal of DNA damage through inactivation of the phosphatase Cdc25 [7]. Checkpoint kinase dependent inhibition of Cdc25 leads to accumulation of phosphorylated cyclin dependent kinases, which prevents cell cycle progression [7]. In bacteria, the SOS-dependent DNA damage checkpoint relies on expression of a cell division inhibitor, though the type of inhibitor varies between bacterial species.

In *Escherichia coli*, the SOS-dependent DNA damage checkpoint is the best understood bacterial checkpoint [6]. Upon activation of the SOS response, the cytoplasmic cell division inhibitor SulA is expressed [10]. SulA accumulation leads to a block in septum formation by preventing the assembly of the cytokinetic ring by FtsZ, a homolog of eukaryotic tubulin [11, 12]. SulA binds directly to FtsZ [13] and inhibits FtsZ polymerization [14, 15]. Recovery from the SulA-induced checkpoint occurs through proteolysis of SulA. Lon is the primary protease responsible for clearing SulA [16–18], although ClpYQ (HslUV) were found to contribute to SulA degradation in the absence of Lon [19–21]. Thus, the mechanisms of DNA damage checkpoint activation by the cytoplasmic protein SulA and subsequent recovery are well understood in *E*. *coli*. The SulA-dependent checkpoint, however, is restricted to *E*. *coli* and a subset of closely related bacteria. It is becoming increasingly clear that most other bacteria use a DNA damage checkpoint with an entirely different mechanism of enforcement and recovery.

An evolutionarily broad group of bacterial organisms have been shown to use a notably different DNA damage checkpoint mechanism [22–25]. In these Gram-positive and Gram-negative organisms, a small protein with a transmembrane domain is expressed that inhibits cell division without targeting FtsZ. One example is in the Gram-negative bacterium *Caulobacter crescentus*, where the SidA and DidA proteins bind to the essential membrane bound divisome components, FtsW/N that contribute to peptidoglycan remodeling [22, 26]. Another example is the Gram-positive bacterium *Bacillus subtilis* in which the SOS-dependent cell division inhibitor is YneA [23]. YneA contains an N-terminal transmembrane domain with the majority of the protein found in the extracellular space [27]. Upon SOS activation, LexA-dependent repression of *yneA* is relieved and *yneA* is expressed [23]. Increased expression of *yneA* results in cell elongation, though FtsZ ring formation still occurs [27], suggesting YneA inhibits cell division through a mechanism distinct from that of SulA. Further investigation found that overexpressed YneA is released into the medium, and that full length YneA is likely the active form of the protein [27]. The mechanism(s) responsible for YneA inactivation is unknown. Therefore, although the use of a small, membrane bound cell division inhibitor is wide-spread among bacteria, in all cases studied the mechanism of checkpoint recovery remains unknown [22–26].

We report a set of forward genetic screens to three different classes of DNA damaging agents using transposon mutagenesis followed by deep sequencing (Tn-seq). Our screen identified two proteases, YlbL and CtpA, that are important for growth in the presence of DNA damage. Mechanistic investigation demonstrates that YlbL and CtpA have overlapping functions, and in the absence of these two proteases, DNA damage-dependent cell elongation is increased and checkpoint recovery is slowed. A proteomic analysis identified accumulation of YneA in the double protease mutant. We also found that DNA damage sensitivity of protease mutants depends solely on *yneA*. Further, we show that both proteases interact with full length YneA in a bacterial two-hybrid assay, and that CtpA is able to digest YneA in a purified system. With these results, we present a model of DNA damage checkpoint recovery for bacteria that use the more wide-spread mechanism employing a small, membrane bound cell division inhibitor.

## Results

### Forward genetic screen rationale and analysis

**Fig 1:**
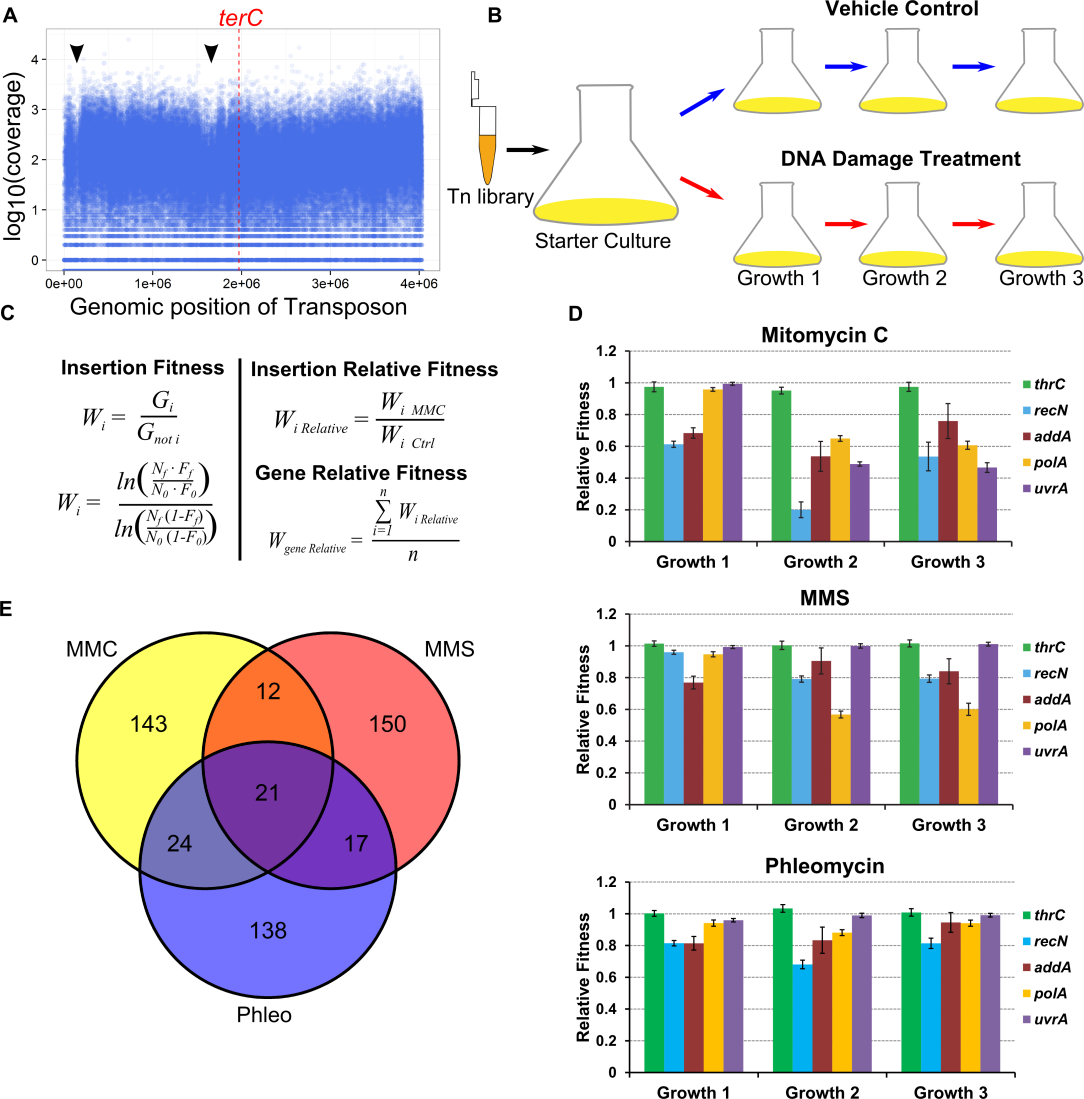
Forward genetic screen experimental design and data analysis. **(A)** A plot of the log_10_ of insertion coverage on the y-axis and genomic position in nucleotides on the x-axis. **(B)** Experimental design for Tn-seq experiments. The transposon library was used to inoculate starter cultures to allow cultures to reach exponential phase. Cultures were split into control and treatment and grown for three growth periods. **(C)** Equations used to calculate relative fitness (W, fitness; G, generations, N, number of cells at the start (N_0_) or end (N_f_) of growth period; F, insertion frequency at the start (F_0_) or end (F_f_) of growth period; n, number of insertions used to calculate average). **(D)** The mean gene relative fitness is plotted as a bar graph for the genes indicated for all three Tn-seq experiments, error bars represent the 95% confidence interval. **(E)** A Venn diagram depicting overlaps of the 200 genes with the lowest fitness and an adjusted p-value less than 0.01 for all three Tn-seq experiments in growth period two.

In order to better understand the DNA damage response in bacteria, we performed three forward genetic screens using *B*. *subtilis*. We generated a transposon insertion library consisting of more than 120,000 distinct insertions (S1 Table). The coverage of each transposon mutant in the library was plotted against the genome coordinates, which showed that the distribution of insertions was approximately uniform across the chromosome in the population of mutants (Fig 1A). Two small exceptions were detected where coverage decreased. Decreased coverage corresponds to regions where many essential genes are clustered (Fig 1A, arrow heads). With the goal of identifying mutants important for the DNA damage response, we grew parallel cultures of either control or DNA damage treatment over three growth periods, modelling our experimental design after a previous report [28; Fig 1B]. Mitomycin C (MMC), methyl methane sulfonate (MMS), and phleomycin (Phleo) were chosen for screening because these agents represent three different classes of antibiotics that damage DNA directly. MMC causes inter- and intra-strand crosslinks and larger adducts [29, 30], MMS causes smaller adducts consisting of DNA methylation [31], and Phleo results in single and double stranded breaks [32, 33]. As a result, we reasoned that the combined data would provide a collection of genes that are generally important for the DNA damage response.

After sequencing, we performed quality control analysis. First, given that sequencing data are count data, the distribution of the coverage should be log-normal [34]. Indeed, the distribution of each replicate for the initial library and starter culture samples is approximately log-normal (S1A Fig). We also found that the distributions for the remaining time points of the pooled replicates followed an approximate log-normal distribution (S1B Fig). The sequencing data and viable cell count data (S1 Table) were used to calculate the fitness of each insertion mutant in each condition [Fig 1C; 35, 36]. The relative fitness of each insertion was calculated by taking the ratio of treatment to control (Fig 1C), thereby isolating fitness effects of the treatments. The relative fitness of each gene was determined by averaging the relative fitness calculated for each insertion within a gene (Fig 1C). To verify that a t-test would be appropriate for determining relative fitness deviating significantly from one, we plotted the distribution of insertion relative fitness. All the distributions were normal with a mean close to one (S1C Fig). We determined the relative fitness for every gene with sufficient data (see supplemental methods), and report the relative fitness values and the adjusted p-values [37] in S2 Table.

### Tn-seq identified genes involved in DNA repair and genes of unknown function

Initial inspection found that several genes known to be involved in DNA repair (*recA*, *ruvAB*, *recN*, and *recOR* [38]) had decreased relative fitness in growth period one of all experiments (S2 Table). A closer analysis of *recN*, *addA*, and *polA*, three genes that are found toward the top of the lists in all treatments, showed that relative fitness is less than one in most cases, though in the Phleo experiment, it appears that the cultures were adapting to the treatment by growth period three (Fig 1D). For comparison, we also plotted the relative fitness of *thrC*, a gene involved in threonine biosynthesis, and found the relative fitness to be about one in all conditions examined (Fig 1D). Importantly, insertion in *uvrA*, a component of the nucleotide excision repair machinery [38, 39] which helps repair MMC adducts but not MMS or Phleo related damage [40, 41], decreased relative fitness in growth periods 2 and 3 with MMC, but did not significantly decrease relative fitness in MMS or Phleo (Fig 1D and S2 Table). Taken together, these results validate the approach by demonstrating that we were able to identify genes known to be involved in DNA repair.

We also wondered whether our results contained false positives. To test this, we decided to experimentally validate the genes with the forty lowest relative fitness values from growth period two in the MMC experiment. We found that eight of the forty gene deletions were not sensitive to MMC in a spot titer assay (Table 1). Several genes that were false positives are located in the genome near genes with validated phenotypes, suggesting that polar effects explain some of the false positives (Table 1; see supplemental results for detailed analysis). To identify genes required generally as part of the DNA damage response, we examined the 200 genes from growth period two with the lowest relative fitness and an adjusted p-value less than 0.01 from all three experiments (S3 Table). We found that 21 genes overlapped for all three experiments (Fig 1E), some of which are known to be involved in DNA repair (*recN*, *addB*, *polA*, *radA*), while several genes have no known function (*e*.*g*., *ylbL* and *ctpA*) (S3 Table).

**Table 1:** Tn-seq yields many false positive results. The forty genes with the lowest relative fitness in the second growth period of MMC Tn-seq experiment are listed. Each gene was deleted and the deletion mutants were tested for sensitivity to MMC using a spot titer assay and a range of MMC concentrations. Genes labeled as not sensitive had no difference in growth relative to the WT strain on MMC containing media, with the exception of *ylbK*, which resulted in a polar effect on *ylbL* (see S3 Fig).

Gene	Mean Fitness	Annotated function	Validation	Proximal gene
***recN***	0.200	Homologous Recombination	[64]; this study	
***sepF***	0.400	Cell Division	This study	
***uvrB***	0.409	Nucleotide excision repair	This study	
***uvrC***	0.484	Nucleotide excision repair	This study	
***uvrA***	0.489	Nucleotide excision repair	[40]; this study	
***addA***	0.537	Homologous Recombination	[65]	
***rnhC***	0.544	RNase HIII	This study	
***crh***	0.565	regulation of carbon metabolism	This study	
***ruvB***	0.568	Homologous Recombination	[66]; this study	
***ylbL***	0.581	putative lon-like protease	This study	
***ponA***	0.587	Peptidoglycan glycosyl transferase	This study	
***bcrC***	0.595	UPP phosphatase	This study	
***ecsA***	0.603	ABC transporter	This study	
***recR***	0.606	Homologous Recombination	[67]; this study	
***queA***	0.612	S-adenosylmethionine tRNA ribosyltransferase-isomerase	Not Sensitive	downstream of *ruvAB*
***addB***	0.622	Homologous Recombination	[68]	
***ecsB***	0.627	ABC transporter	This study	
***polA***	0.649	DNA polymerase I	[40]	
***ylmG***	0.656	hypothetical protein	Not sensitive	downstream of *sepF*
***lgt***	0.661	lipomodification of lipoproteins	Not sensitive	
***ctpA***	0.673	c-terminal processing protease	This study	
***ripX***	0.674	Homologous Recombination	[69]; this study	
***ytmP***	0.690	putative kinase/phosphotransferase	This study	
***recG***	0.695	Homologous Recombination	[69]; this study	
***ydzU***	0.714	unknown	This study	
***walH***	0.715	Regulation of cell wall metabolism	This study	
***ylmE***	0.718	hypothetical protein	Not sensitive	upstream of *sepF*
***sdaAB***	0.724	L-serine dehydratase	Not sensitive	upstream of *recG*
***ysoA***	0.725	putative hydrolase	This study	
***recX***	0.729	Homologous Recombination	[70]	
***walJ***	0.729	Regulation of cell wall metabolism	This study	
***recD2***	0.731	Homologous Recombination	[41]	
***radA***	0.742	Homologous Recombination	This study	
***ylbK***	0.746	putative phospholipase	Not sensitive	upstream of *ylbL*
***sodA***	0.763	super-oxide dismutase	This study	
***cymR***	0.770	regulation of sulfur metabolism	Not sensitive	upstream of *recD2*
***yprA***	0.773	putative helicase	This study	
***yprB***	0.777	putative DnaQ-like exonuclease	This study	
***ywrC***	0.785	regulation of chromate export	Not sensitive	
***yycI***	0.790	Regulation of cell wall metabolism	This study	

### YlbL and CtpA require putative catalytic residues for function

**Fig 2:**
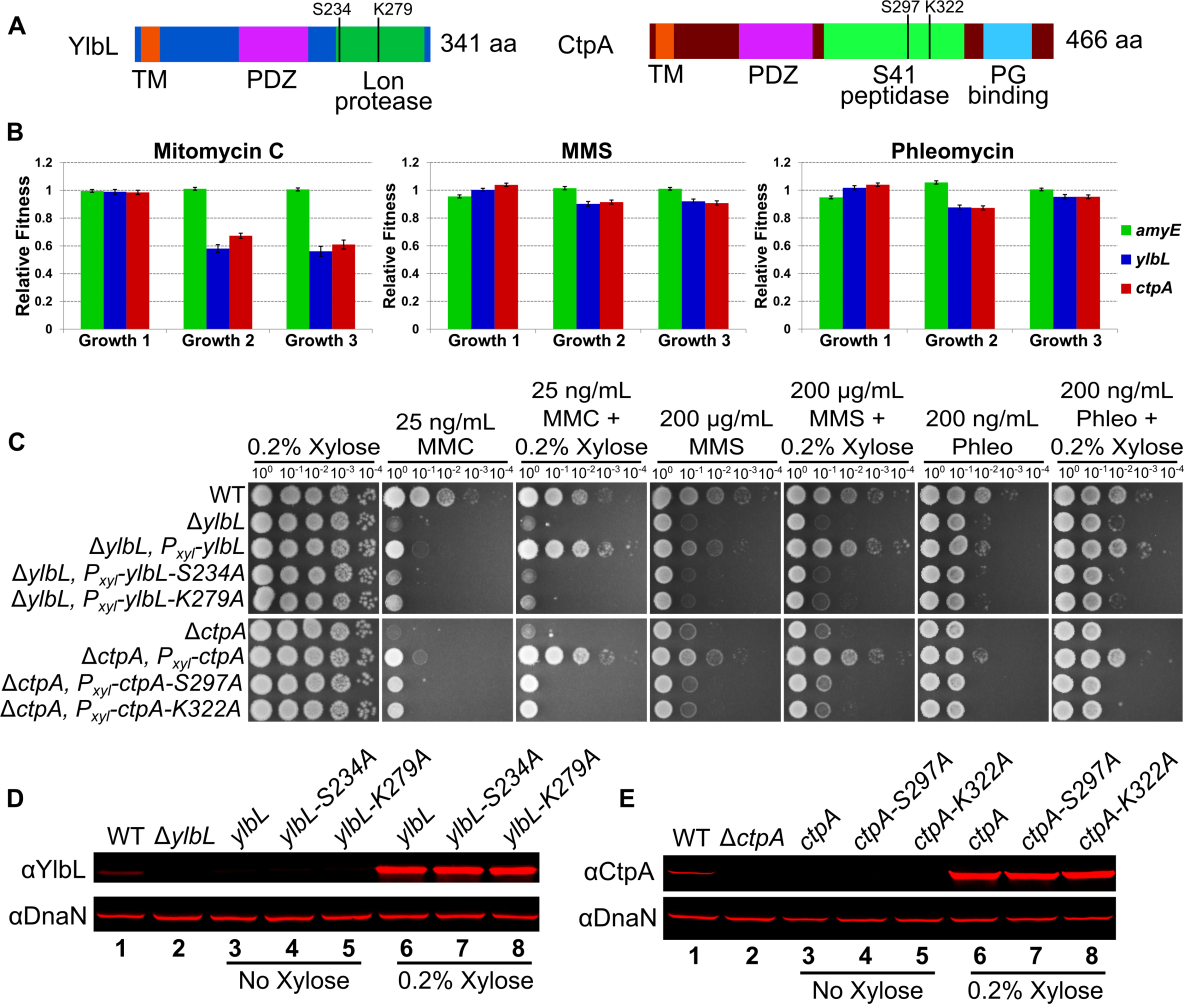
YlbL and CtpA require putative catalytic residues for function. **(A)** Schematics of YlbL and CtpA proteins depicting the domain organization. **(B)** The mean gene relative fitness is plotted as a bar graph for *amyE*, *ylbL*, and *ctpA* for all three Tn-seq experiments, error bars represent the 95% confidence interval. **(C)** Spot titer assays using the genotypes indicated and plated on LB agar media containing the indicated drugs. **(D)** Western blot analysis of cell lysates from the indicated genotypes grown with or without xylose using antiserum against YlbL (upper panel) and DnaN (lower panel). **(E)** Western blot analysis of cell lysates from the indicated genotypes grown with or without xylose using antiserum against CtpA (upper panel) and DnaN (lower panel).

Among the genes important for growth in the presence of DNA damage, we focused on two putative proteases YlbL and CtpA. YlbL is predicted to have three domains: a transmembrane domain, a Lon protease domain, and a PDZ domain (Fig 2A). CtpA is predicted to have four domains: a transmembrane domain, a S41 peptidase domain, a PDZ domain, and a C-terminal peptidoglycan (PG) binding domain (Fig 2A). In all three Tn-seq experiments, the relative fitness of insertions in either *ylbL* or *ctpA* was significantly less than one in the second and third growth periods (Fig 2B), suggesting that absence of either protease results in sensitivity to DNA damage. In contrast, a control gene *amyE*, which is involved in starch utilization, had a relative fitness of approximately one in all conditions examined (Fig 2B). To verify the Tn-seq results, we constructed clean deletions of *ylbL* and *ctpA* and found both mutants to be sensitive to DNA damage in a spot titer assay (Fig 2C). Each phenotype was also complemented by ectopic expression of each protease in its respective mutant background (Fig 2C). To identify putative catalytic residues, we aligned the protease domain of YlbL to LonA and LonB from *B*. *subtilis* and Lon from *E*. *coli*. The sequence alignment revealed that YlbL contains a putative catalytic dyad consisting of a serine (S234) and a lysine (K279) (S2A Fig). Similarly, we aligned CtpA to its homologs CtpB from *B*. *subtilis* and Prc from *E*. *coli*, which identified a putative catalytic triad consisting of a serine (S297), a lysine (K322), and a glutamine (Q326) (S2B Fig). To test whether these putative catalytic residues were required for function, we attempted to complement the DNA damage sensitivity phenotype via ectopic expression of serine and lysine mutants. Both the serine and lysine mutants of YlbL and CtpA failed to complement the deletion phenotypes (Fig 2C). The variants and the wild-type proteases were ectopically expressed to the same level *in vivo* (Fig 2D), suggesting that the lack of complementation is not due to instability caused by the amino acid changes. With these results, we conclude that protease activity is required for YlbL and CtpA to function in response to DNA damage.

### YlbL and CtpA have overlapping functions

**Fig 3:**
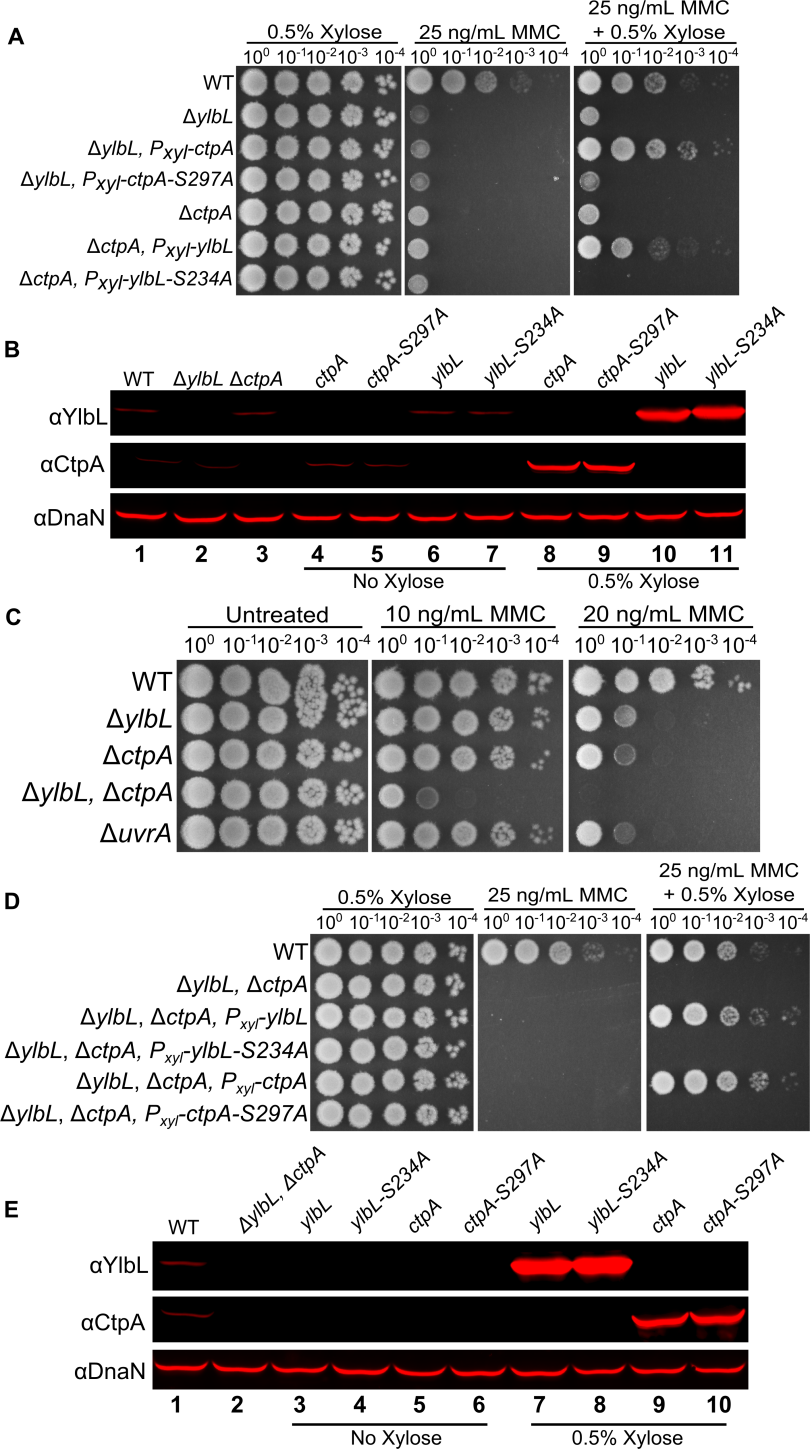
YlbL and CtpA have overlapping functions. **(A)** Spot titer assay using the indicated genotypes and media. **(B)** Western blot analysis of cell lysates from the genotypes in panel A, using the indicated antiserum. **(C & D)** Spot titer assay using the indicated genotypes and media. **(E)** Western blot analysis of cell lysates from the genotypes indicated in panel D, using the indicated antiserum.

The similarity in phenotypes led us to hypothesize that YlbL and CtpA have overlapping functions. To test this, we performed a cross-complementation experiment using spot titer assays for MMC sensitivity. Over-expression of YlbL, but not YlbL-S234A, complemented a *ctpA* deletion (Fig 3A & 3B). Similarly, over-expression of CtpA, but not CtpA-S297A, complemented a *ylbL* deletion (Fig 3A & 3B). In addition, deletion of both proteases rendered *B*. *subtilis* hypersensitive to MMC, even more so than loss of *uvrA*, which codes for the protein responsible for recognizing MMC adducts as part of nucleotide excision repair [Fig 3C; 38, 39]. To further test the hypothesis that YlbL and CtpA have overlapping functions, we over-expressed each of the proteases separately in the double protease mutant background and observed a complete rescue of MMC sensitivity upon expression of the wild type (WT), but not the serine variants (Fig 3D & 3E).

### DNA damage-dependent cell division delay is increased in protease deletions

The experiments performed thus far cannot distinguish between sensitivity to MMC resulting from cell death, growth inhibition or both. To determine whether sensitivity arises from cell death, we performed a survival assay using an acute treatment of MMC. We detected a slight decrease in percent survival as MMC concentration increased in the Δ*ylbL* and the double mutant strain (S4A Fig). We compared the decrease in percent survival in single and double protease mutants to a Δ*uvrA* strain, which has been shown previously to be acutely sensitive to MMC [40]. The strain lacking *uvrA* was very sensitive to an acute treatment of MMC (S4A Fig), whereas, the double protease deletion strain was significantly less sensitive to acute exposure compared with Δ*uvrA* (compare Figs 3C & S4A). Taken together, we conclude that MMC sensitivity of the protease mutants observed in spot titer assays is primarily caused by growth inhibition.

**Fig 4:**
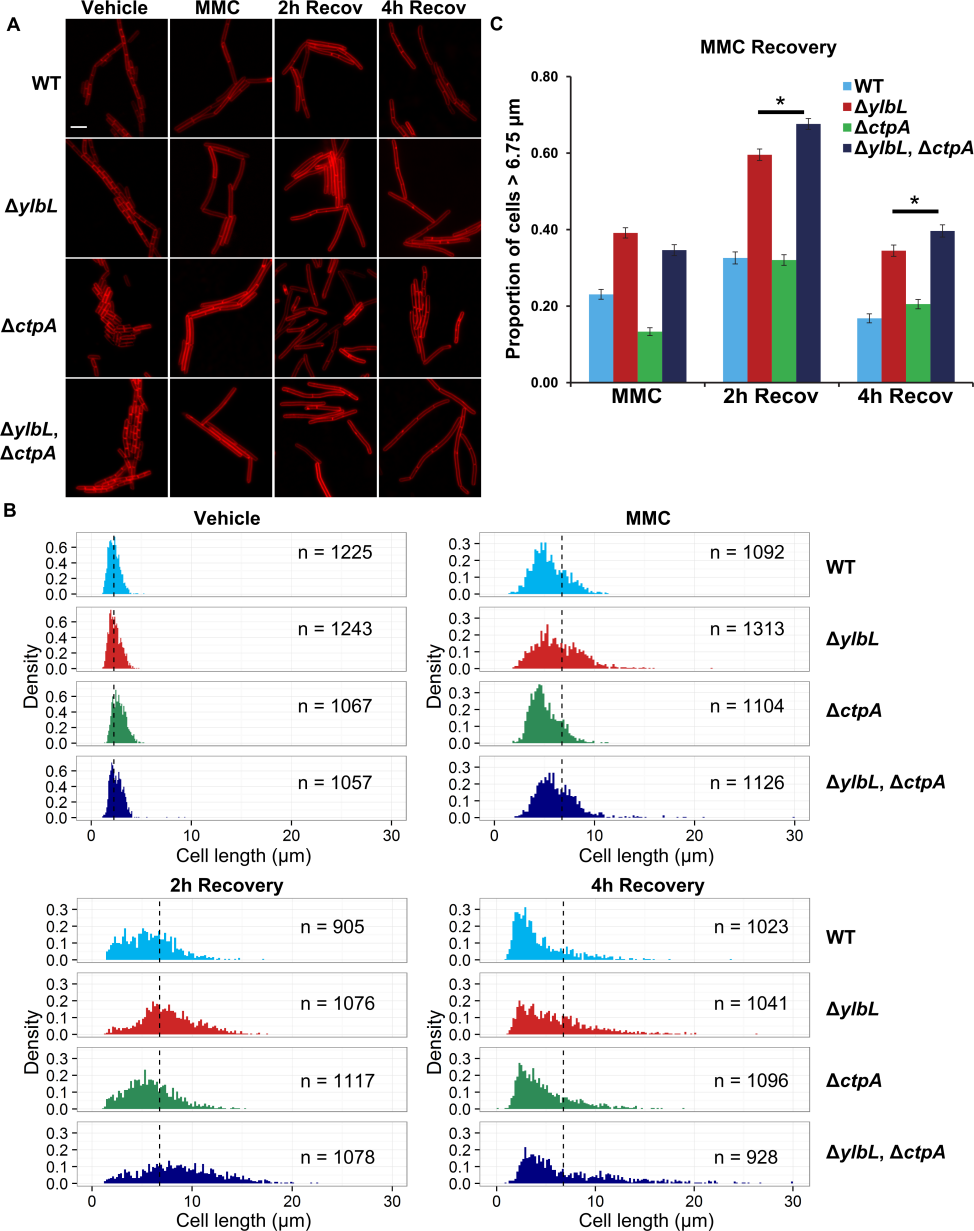
DNA damage delays cytokinesis in cells with protease deletions. **(A)** Representative micrographs of cells with the indicated genotypes at the indicated time points. Membranes were stained with FM4-64. Scale bar is 5 μm. **(B)** Cell length distributions plotted as histograms. The y-axis in all graphs is normalized by the sample size yielding the density, and the x-axis is the cell length in μm. The number of cells scored in each distribution is indicated as “n =” and the genotype of each strain is indicated. The dotted vertical line in the “Vehicle” distributions is plotted at 2.25 μm, the approximate mean for all strains. The dotted vertical line in the remaining distributions is at 6.75 μm, which is three times the average length of untreated cells. **(C)** The proportion of cells represented by the histograms in panel B with length greater than 6.75 μm is plotted as a bar graph. The error bars represent the standard deviation, $\sqrt{\frac{p(1-p)}{n}}$, where p represents the proportion and n is the sample size. The asterisk indicates a p-value less than 0.05.

We hypothesized that sensitivity to DNA damage resulting from growth inhibition could also be explained by inhibiting cell proliferation, or by inhibiting cell division rather than cell growth. To distinguish between these two possibilities, we measured cell length, because inhibition of proliferation should be observed as an increase in cell length, consistent with a failure in checkpoint recovery. Thus, we designed a MMC recovery assay, reasoning that following treatment with MMC, cells lacking YlbL, CtpA, or both, would remain elongated showing slower checkpoint recovery relative to the WT strain. We grew cultures either in a vehicle control or in the presence of MMC. After a two-hour treatment, the MMC containing media was removed and cells were washed. Cells were then transferred to fresh media without MMC and allowed to continue growth to assay for checkpoint recovery. Although cells appeared to be elongated in the Δ*ylbL* and double mutant strains, there was heterogeneity in the population (Fig 4A). As a result, we measured the cell length of at least 900 cells for each genotype and each condition and plotted the cell length distributions as histograms (Fig 4B). There was no difference in the vehicle control cell length distributions (Fig 4B). The MMC treatment of all strains resulted in a rightward shift in the distribution for all strains (Fig 4B, compare upper panels). When comparing the protease deletions to the WT, the difference in distribution could be visualized by considering the percentage of cells greater than 6.75 μm in length, which is about three cell lengths of 2.25 μm each. We found that deletion of *ylbL* resulted in an increase in the percentage of cells longer than 6.75 μm in MMC treated cultures and after both two hours and four hours of recovery (Fig 4C). Deletion of *ctpA*, however, resulted in a very slight, though significant (p-value = 0.0142 for one-tailed Z-test), increased percentage of cells longer than 6.75 μm after 4 hours of recovery (Fig 4C). The double mutant resulted in a percentage of cells slightly greater than Δ*ylbL* alone after both two hours (p-value = 0.0001 for one-tailed Z-test) and four hours (p-value = 0.0088 for one-tailed Z-test) of recovery (Fig 4C).

Taken together, we conclude that YlbL is the primary protease under these conditions, with CtpA also contributing. We also conclude that cells lacking YlbL or both YlbL and CtpA take longer to divide following exposure to MMC, which is consistent with DNA damage sensitivity resulting from inhibition of cell proliferation. Further, the observation of inhibition of cell proliferation suggests that YlbL and CtpA proteases could be important for DNA damage checkpoint recovery (see below).

### YlbL and CtpA levels are not regulated by DNA damage

A potential model to regulate YlbL and CtpA in response to DNA damage is to increase protein levels following exposure to DNA damage. Increased protease levels in response to DNA damage could promote the DNA damage checkpoint recovery when needed. To test this model, we monitored YlbL and CtpA protein levels via Western blotting over the course of the MMC recovery assay. YlbL and CtpA protein levels did not change relative to the loading control DnaN throughout the course of the experiment (S4B & S4C Fig). As a positive control, we performed the same experiment and monitored RecA protein levels and found that, indeed, RecA protein levels increased (S4B & S4C Fig), as expected because *recA* is induced as part of the SOS response [42, 43]. We conclude that YlbL and CtpA protein levels are not regulated by DNA damage.

### The cell division inhibitor YneA accumulates in protease mutants

**Fig 5:**
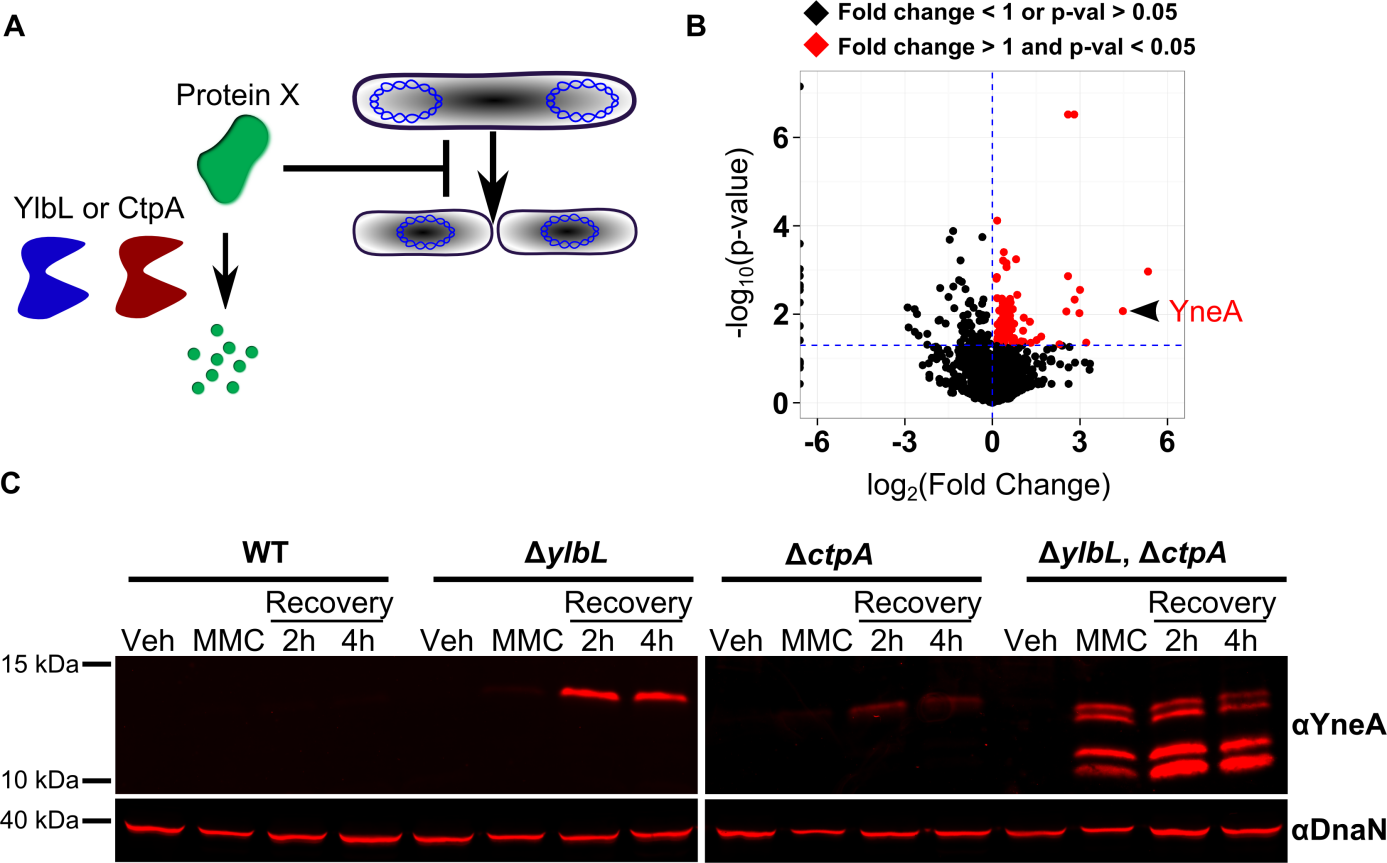
YneA accumulates in protease mutants. **(A)** A model for the function of YlbL and CtpA in regulating cell division. **(B)** Proteomics data plotted as fold change (Double Mutant/WT) vs. the p-value. Points plotted in black have a fold change less than one or a p-value greater than or equal to 0.05, and points plotted in red have a fold change greater than one and a p-value less than 0.05. **(C)** Western blot analysis of cell lysates from strains with the indicated genotypes at the indicated time points from the MMC recovery assay (see methods), using YneA or DnaN antiserum.

The data presented thus far led us to hypothesize that in the absence of YlbL and CtpA, a protein accumulates which results in inhibition of cell division (Fig 5A). To identify the accumulating protein, we performed an analysis of the entire proteome of WT and double protease mutant cell extracts. We chose to analyze the proteomes of cells after two hours of recovery, because the cell length distributions differed most between WT and the double protease mutant (Fig 4B). We found that the normalized spectral count data had similar distributions for both WT and the double mutant, which were approximately log normal (S5A Fig). We verified that the distribution of the test statistic (the difference in double mutant average and WT average) was normally distributed (S5B Fig), thus allowing a t-test to be used. We also performed a principle component analysis and found that WT replicates and double mutant replicates each clustered together (S5C Fig).

In total, 2329 proteins were detected (S4 Table), and 183 proteins were found to be differentially represented (p-value < 0.05) in the double mutant relative to WT (S5 Table). Of the proteins differentially represented in the double mutant, 104 had a fold change greater than one (Fig 5B, red points). There are three major mechanisms that have been reported in *B*. *subtilis* to inhibit cell division: 1) Noc dependent nucleoid occlusion [44], 2) FtsL depletion [45, 46], and 3) expression of YneA [23]. One possibility was that Noc protein levels were higher in the double mutant, but we observed no difference in Noc levels (S5D Fig). Another possibility was that FtsL or the protease RasP, which degrades FtsL, was affected in the protease mutant background [46]. We found no difference in relative protein abundance of FtsL or RasP (S5D Fig), ruling out the FtsL/RasP pathway. Among the top 10 proteins that were more abundant in the double mutant was YneA, the SOS-dependent cell division inhibitor (S5 Table). We asked if the enrichment of YneA was simply because it is SOS induced. We analyzed the relative abundance of several other proteins that are known to be SOS induced, including RecA, UvrA, UvrB, DinB, and YneB [42], which is in an operon with YneA [23]. We found that none of these other proteins were enriched in the double mutant (S5E Fig). These results suggest that YneA accumulation is not a result of increased SOS activation, and regulation of YneA accumulation is likely to be post translational, because the protein levels of another member of the operon, YneB were unchanged. Taken together, our proteomics data suggest that YlbL and CtpA promote DNA damage checkpoint recovery through regulating YneA protein abundance.

We directly tested for YneA accumulation in protease mutants throughout the MMC recovery assay using Western blotting. YneA accumulated in all protease deletion strains after 2 hours and 4 hours of recovery, though YneA accumulation in Δ*ctpA* was slight (Fig 5C). In the double mutant, YneA accumulated in the MMC treatment condition in addition to both recovery time points (Fig 5C). In the double mutant we observed multiple YneA species, which we hypothesize to be the result of unnaturally high YneA protein levels resulting in non-specific cleavage by other proteases. With these results, we suggest that YneA is a substrate of YlbL and CtpA, both of which degrade YneA allowing for checkpoint recovery.

### *yneA* is required for DNA damage sensitivity and cell elongation phenotypes

**Fig 6:**
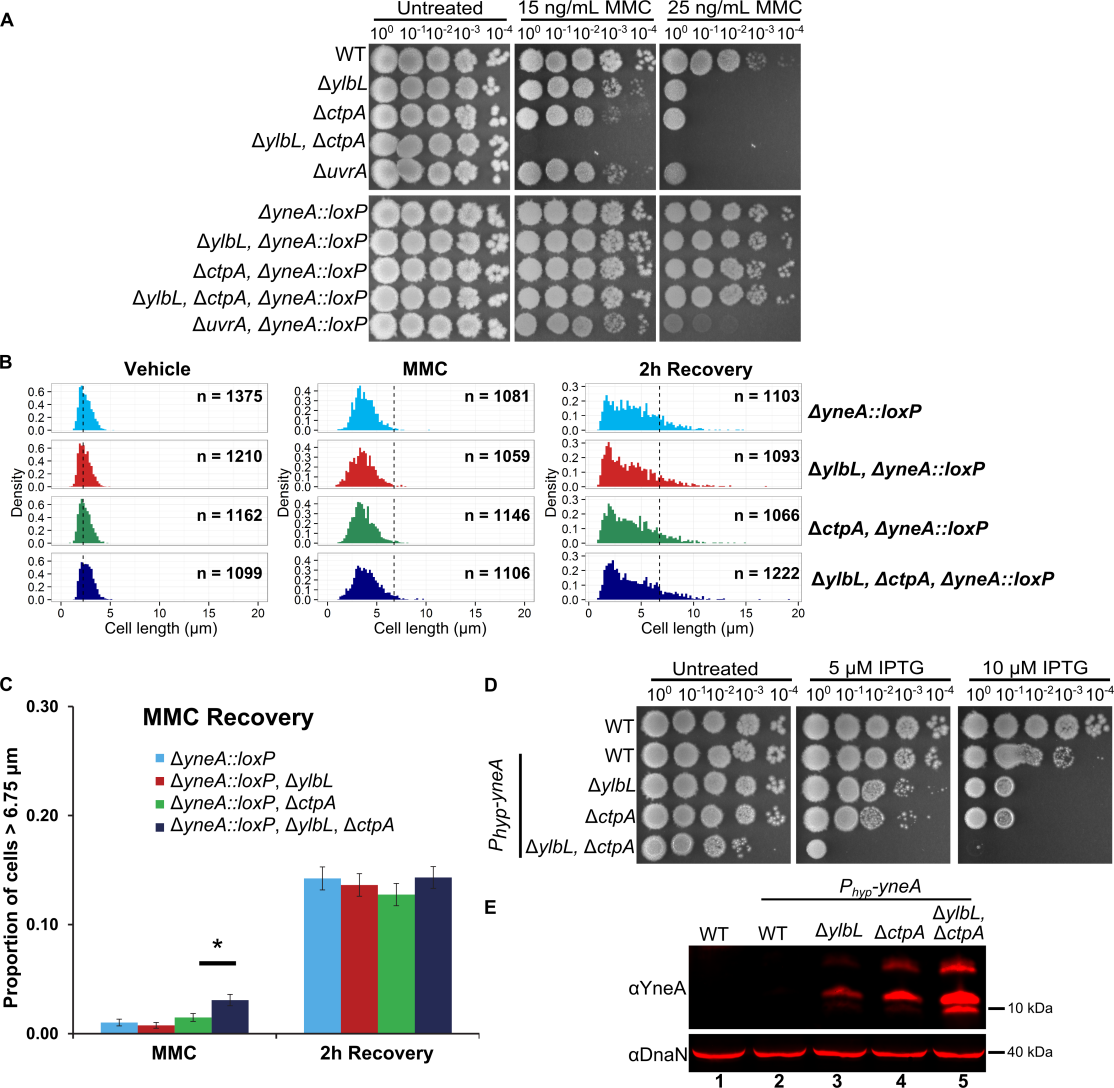
*yneA* is required for DNA damage sensitivity and cell elongation phenotypes. **(A)** Spot titer assay using the indicated genotypes and media. **(B)** Cell length distributions plotted as histograms. The number of cells scored in each distribution is indicated as “n =” and the genotype of each strain is indicated above the distributions. The dotted vertical line in the “Vehicle” distributions is at the approximate mean of 2.25 μm. The dotted vertical line in the remaining distributions is at 6.75 μm. The y-axis in all graphs is normalized by the sample size yielding the density, and the x-axis is the cell length in μm. **(C)** The proportion of cells greater than 6.75 μm from the distributions in panel B is plotted as a bar graph. The error bars represent the standard deviation, $\sqrt{\frac{p(1-p)}{n}}$, where p represents the proportion and n is the sample size. The asterisk indicates a p-value less than 0.05. **(D)** Spot titer assay testing the effect of over production of YneA using the indicated concentration of the inducer IPTG. **(E)** Western blot analysis of cell lysates using 100 μM IPTG for YneA expression with the indicated genotypes, using the indicated antiserum.

Although accumulation of YneA fit our data well, we considered that the other proteins enriched greater than five-fold in the double mutant may have contributed to the DNA damage sensitivity phenotype. To test this, we constructed deletions of each gene in WT and the double mutant and tested for MMC sensitivity. We found that no single deletion of each of the 10 genes resulted in sensitivity to MMC (S6A Fig). In the double mutant, only deletion of *yneA* was able to rescue the sensitivity to MMC (S6A Fig). We verified that deletion of *yneA* could rescue MMC sensitivity in all protease mutant backgrounds (Fig 6A). We examined cell length in the DNA damage recovery assay. As expected, deletion of *yneA* resulted in less severe cell elongation relative to WT (compare WT in Fig 4B and Δ*yneA∷loxP* in Fig 6B). In addition, deletion of *ylbL*, *ctpA*, or both no longer changed the cell length distribution in the absence of *yneA* at the two-hour recovery time point (Figs 6B, 6C and S6B). In the MMC treatment, we did observe a slight increase (p-value = 0.0004 for one-tailed Z-test) in the percentage of cells greater than 6.75 μm in the double protease deletion strain compared to WT (Fig 6C). Given that MMC sensitivity and most cell elongation in protease mutants depends on *yneA*, we hypothesized that expression of YneA alone would be sufficient to inhibit growth to a greater extent in the protease mutants. Indeed, strains lacking YlbL, CtpA or both were more sensitive to over-expression of *yneA* from an IPTG inducible promoter than WT (Fig 6D). Further, we show that YneA accumulated in the protease mutant strains following *yneA* ectopic expression (Fig 6E). We conclude that YneA accumulation results in severe growth inhibition in cells lacking YlbL and CtpA.

### CtpA specifically digests YneA *in vitro*

**Fig 7:**
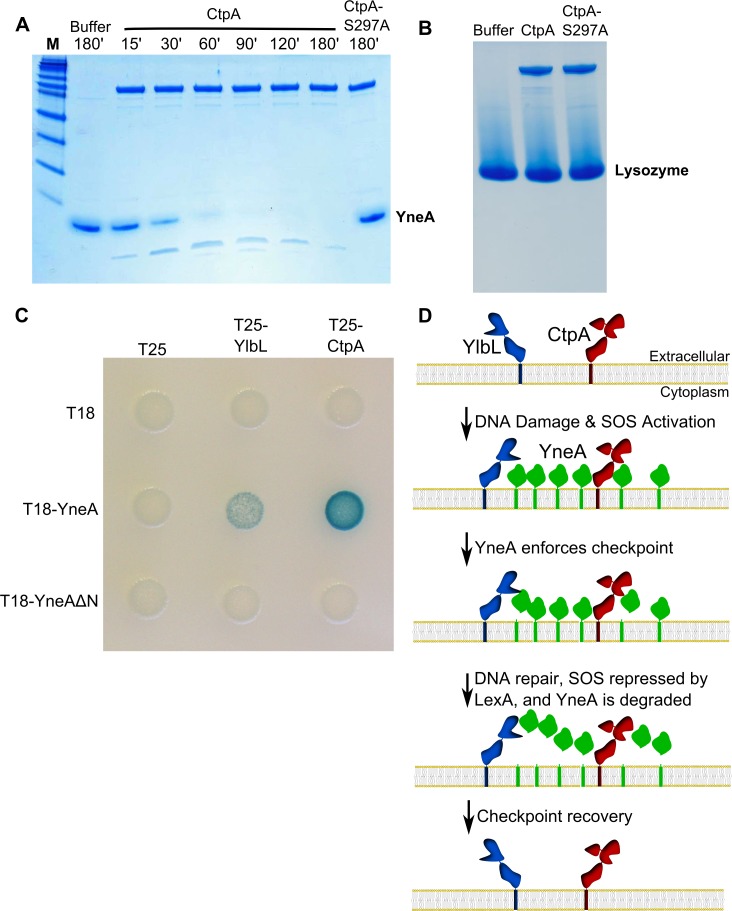
DNA damage checkpoint recovery in *Bacillus subtilis*. **(A)** Protease assay incubating purified CtpA or CtpA-S297A with purified YneA for the indicated time followed by SDS-PAGE and staining with coomassie blue. **(B)** Protease assay incubating purified CtpA or CtpA-S297A with commercially available lysozyme for 3 hours followed by SDS-PAGE and staining with coomassie blue. **(C)** Bacterial two hybrid assay using T18 fusions (rows) and T25 fusions (columns) co-transformed into *E*. *coli*. The T25 plasmids are: the empty vector (T25), T25-YlbL-S234A (T25-YlbL), and T25-CtpA-S297A (T25-CtpA). The T18 plasmids are: the empty vector (T18), T18-YneA, and T18-YneAΔN (which lacks the transmembrane domain). **(D)** A model for YlbL- and CtpA-dependent DNA damage checkpoint recovery. YlbL and CtpA are present as membrane proteases, and when high amounts of DNA damage are present, YneA production overwhelms both proteases resulting in delayed cell division. After DNA repair is complete and YneA expression decreases, YneA is cleared and cell division proceeds.

To test the hypothesis that YneA is a direct substrate of the proteases we purified YneA (a.a. 28–103), CtpA (a.a 38–466), and YlbL (a.a 36–341) lacking their N-terminal transmembrane domains to allow for isolation. We were unable to detect protease activity from YlbL using YneA, lysozyme, or casein as substrates (S7 Fig; see discussion). When purified CtpA was incubated with YneA, we observed digestion of YneA over time, but no digestion was observed using CtpA-S297A (Fig 7A). To test if CtpA activity against YneA was specific we completed the same reaction using lysozyme as a substrate and detected no activity (Fig 7B). We conclude that YneA is a direct and specific substrate of CtpA.

### YlbL-S234A and CtpA-S297A interact with YneA

Although we could not detect YlbL protease activity *in vitro* we asked if YlbL could interact with YneA. To test this, we used a bacterial two-hybrid assay [47, 48]. We used this assay because it is effective at detecting interactions between membrane proteins [22, 26, 49]. We tested YlbL-S234A and CtpA-S297A to prevent digestion of YneA, and assayed for interaction with full length YneA or YneA without its transmembrane domain (YneAΔN) as a control. We found that YlbL and CtpA both interacted with full length YneA (Fig 7C), but no interaction was detected with YneAΔN (Fig 7C), likely due to YneA failing to localize to the membrane. Given that YlbL did not have activity *in vitro* and we detected an interaction with YneA in the bacterial two-hybrid assay, we suggest that YneA is a direct substrate of YlbL and that YlbL requires full length YneA for interaction.

## Discussion

How do YlbL and CtpA recognize YneA as a substrate? An intriguing facet of this checkpoint recovery mechanism is the use of unrelated proteases. YlbL and CtpA both have transmembrane domains and PDZ domains, but the peptidase domains are very different. CtpA has a S41 peptidase domain and is homologous to Tail-specific protease or Tsp (also Prc), which recognizes the C-terminus of its substrate through its PDZ domain [50, 51]. We suggest that CtpA also recognizes YneA through the PDZ domain and that this mechanism explains how CtpA recognizes its other cognate substrates. In fact, the study by Mo and Burkholder identified a residue at the C-terminus of YneA (D97) which when mutated to alanine stabilizes YneA [27]. It is tempting to speculate that D97 in YneA is important for CtpA to recognize YneA. The mechanism by which YlbL recognizes YneA is less clear. YlbL has a unique domain organization not found in other studied proteases. YlbL does not have the AAA+ ATPase domain common in other Lon proteases, which is logical given that YlbL likely resides extracellularly in order to degrade YneA. Instead of an ATPase domain, YlbL has a PDZ domain, which could act as a substrate recognition domain or as an inhibitory domain similar to the PDZ domain of DegS [52–54]. The bacterial two hybrid assay suggests that YlbL does recognize YneA directly (Fig 7C), though we cannot rule out the possibility that there is an adaptor protein that recognizes YneA when these proteins are tethered to the membrane. We also did not identify a potential adaptor in the Tn-seq data further suggesting a direct interaction does occur between YlbL and YneA. A final possibility is that YlbL recognizes YneA through the transmembrane domain, which then activates the Lon peptidase domain to degrade or cleave YneA. This would also explain the reason we were unable to detect activity using YlbL lacking its transmembrane domain. In any case, further experiments are necessary to elucidate the mechanism by which YlbL recognizes YneA.

All organisms control cellular processes through regulated signaling. To regulate a cellular process a signaling pathway must have mechanisms of activation and inactivation. Many bacteria use a small membrane protein as an SOS-induced DNA damage checkpoint protein [22–25]. The mechanism of checkpoint recovery, however, for organisms using membrane protein checkpoints has remained unclear. Our comprehensive study identified a dual protease mechanism of DNA damage checkpoint recovery (Fig 7D). Proteases YlbL and CtpA are constitutively present in the plasma membrane of cells even in the absence of DNA damage. After encountering DNA damage, YneA expression is induced. We hypothesize that YlbL and CtpA activities become saturated by increased YneA expression, which results in a delay of cell division. Following DNA repair, expression of YneA decreases and YlbL and CtpA clear any remaining YneA allowing cell division to resume.

DNA damage checkpoints are of fundamental importance to biology, and we have discovered the pathway responsible for checkpoint inactivation and cell cycle re-entry in *B*. *subtilis*. These findings represent an important advance in identifying how checkpoint recovery occurs in bacteria. The membrane bound cell division inhibitors identified to date [22–25], are not homologs of YneA, and in fact the only unifying feature is that they are small membrane bound proteins [22–25]. This poses a great challenge because the components of checkpoint enforcement and recovery need to be experimentally identified. Our study serves as a model to identify the checkpoint recovery proteases through forward genetics, which in turn could be used to identify the enforcement protein through proteomics. The critical feature of our approach was the use of several growth periods in Tn-seq, which allowed us to identify both proteases. Thus, we propose a strategy using the combined approaches of forward genetics and targeted proteomics to identify the DNA damage checkpoint pathways in genetically tractable bacterial pathogens.

Recovery from a DNA damage checkpoint is a critical process for all organisms. One theme found throughout biology is the use of multiple proteins with overlapping functions. In eukaryotes, the phosphorylation events that establish the checkpoint are removed by multiple phosphatases [55, 56]. In *E*. *coli*, there are two cytoplasmic proteases, Lon and ClpYQ, that have been found to degrade the cell division inhibitor SulA [16, 18–20]. Our study further extends the use of multiple factors in regulating checkpoint recovery to *B*. *subtilis*, by describing a mechanism using two proteases. In eukaryotes, multiple proteins with overlapping functions often exist due to spatial or temporal restrictions, which appears to at least partially explain the use of multiple factors in checkpoint recovery [55, 56]. In *E*. *coli*, ClpYQ was found to be important at higher temperatures in the absence of Lon [19], again suggesting that each protease functions under specific conditions. In the case of YlbL and CtpA, however, there appears to be a shared responsibility in rich media. Deletion of each protease results in DNA damage sensitivity and the double mutant has a more severe sensitivity. In contrast, during growth in minimal media, YlbL appears to be the primary protease, as the cell elongation phenotype is more pronounced in cells lacking *ylbL*. Still it is unclear how or when each protease functions. Why isn’t one protease sufficient to degrade YneA? Do the proteases occupy distinct loci in the cell, requiring that each protease degrades a specific YneA pool? Another possibility is that protease levels are constrained by another evolutionary pressure, such as substrates unique to each protease. Thus, the cell cannot maintain the individual proteases at levels required to titrate YneA as part of the DDR, because the levels of another substrate would be too low. Another explanation is that using multiple factors is an evolutionary strategy that increases the fitness of an organism. It is clear that checkpoint recovery is crucial, because the fitness of cells lacking *ylbL* or *ctpA* is significantly decreased in the presence of DNA damage (S2 Table).

Although the dual protease mechanism described here resolves an important step in the DDR, our data also reveal the complexity of the system. After we exposed cells to MMC the cells elongated. We noticed however, that not all elongation depended on *yneA* (see Fig 6B), suggesting another mechanism for cell cycle control. In *B*. *subtilis*, there have been reports of *yneA*-independent control of cell division following replication stress [45, 57, 58]. The essential cell division component FtsL has been reported to be unstable and depletion leads to inhibition of cell division [58]. Further, *ftsL* transcript levels were reported to decrease following replication stress independent of the SOS response [45], thus linking depletion of the unstable FtsL protein to cell division control following replication stress. A study using a replication block consisting of the Tet-repressor bound to a Tet-operator array, observed cell division inhibition independent of *yneA*, *noc*, and FtsL [57]. Interestingly, recent studies of *Caulobacter crescentus* uncovered two cell division inhibitors that are expressed in response to DNA damage, with one inhibitor SOS-dependent and the other SOS-independent [22, 26]. In *B*. *megaterium*, a recent study found that the transcript of *yneA* is unstable following exposure to DNA damage [59], suggesting yet another layer of regulation. No factor was identified to regulate *yneA* transcripts in the previous study, though it is possible that one of the genes of unknown function identified in our screens could regulate *yneA* mRNA. Together, these studies highlight the complexity of regulating the DNA damage checkpoint in bacteria.

## Materials and methods

### Bacteriological methods and chemicals

Bacterial strains, plasmids, and oligonucleotides used in this study are listed in S6 Table and the construction of strains and plasmids is detailed in the supplemental methods. All *Bacillus subtilis* strains are isogenic derivatives of PY79 [60]. *Bacillus subtilis* strains were grown in LB (10 g/L NaCl, 10 g/L tryptone, and 5 g/L yeast extract) or S7_50_ minimal media with 2% glucose (1x S7_50_ salts (diluted from 10x S7_50_ salts: 104.7g/L MOPS, 13.2 g/L, ammonium sulfate, 6.8 g/L monobasic potassium phosphate, pH 7.0 adjusted with potassium hydroxide), 1x metals (diluted from 100x metals: 0.2 M MgCl_2_, 70 mM CaCl_2_, 5 mM MnCl_2_, 0.1 mM ZnCl_2_, 100 μg/mL thiamine-HCl, 2 mM HCl, 0.5 mM FeCl_3_), 0.1% potassium glutamate, 2% glucose, 40 μg/mL phenylalanine, 40 μg/mL tryptophan) at 30°C with shaking (200 rpm). Mitomycin C (MMC), methyl methane sulfonate (MMS), and phleomycin were used at the concentrations indicated in the figures. The following antibiotics were used for selection in *B*. *subtilis* as indicated in the method details: spectinomycin (100 μg/mL), chloramphenicol (5 μg/mL), and erythromycin (0.5 μg/mL). Selection of *Escherichia coli* (MC1061 or TOP10 cells for cloning or BL21 for protein expression) transformants was performed using the following antibiotics: spectinomycin (100 μg/mL) or kanamycin (50 μg/mL).

### Tn-seq

A transposon insertion library was constructed similar to [61] with modifications described in the supplemental methods. Tn-seq experiments were designed with multiple growth periods similar to a prior description [28], with a detailed description in the supplemental methods. Sequencing library construction and data analysis were performed as described previously [35, 61] with modifications described in the supplemental methods. Sequencing data were deposited into the GEO database with accession number GSE109366.

### Spot titer assays

*B*. *subtilis* strains were struck out on LB agar and incubated at 30°C overnight. The next day, a single colony was used to inoculate a 2 mL LB culture in a 14 mL round bottom culture tube, which was incubated at 37°C on a rolling rack until OD_600_ was 0.5–1. Cultures were normalized to OD_600_ = 0.5 and serial diluted. The serial dilutions were spotted (4 μL) on the agar media indicated in the figures and the plates were incubated at 30°C overnight (16–20 hours). All spot titer assays were performed at least twice.

### Survival assays

Survival assays using an acute treatment of mitomycin C were performed as previously described [62]. Cultures were grown to an OD_600_ of about 1, and triplicate samples of 0.6 mL of an OD_600_ = 1 equivalent were taken and cells were pelleted via centrifugation: 10,000 *g* for 5 minutes at room temperature (all subsequent centrifugation steps were identical). Cells were washed with 0.6 mL 0.85% NaCl (saline) and pelleted via centrifugation. Cell pellets were re-suspended in 0.6 mL saline, and 100 μL aliquots were distributed for each MMC concentration. MMC was added to each tube to yield the final concentration stated in the figure in a total volume of 200 μL, and cells were incubated at 37°C for 30 minutes. Cells were pelleted via centrifugation to remove MMC, re-suspended in saline, and a serial dilution yielding a scorable number of cells (about 30–300) was plated on LB agar to determine the surviving fraction of cells. Each experiment was performed three times in triplicate for each strain.

### Antiserum production

Purified proteins (see supplemental methods for purification protocols) were submitted to Covance for antibody production using rabbits. Two rabbits were used in the 77 day protocol, and the serum with the least background was used for Western blots.

### Western blotting

For YlbL, CtpA, RecA, and DnaN Western blots, a cell pellet equivalent of 1 mL OD_600_ = 1 was re-suspended in 100 μL 1x SMM buffer (0.5 M sucrose, 0.02 M maleic acid, 0.02 M MgCl_2_, adjusted to pH 6.5) containing 1 mg/mL lysozyme and 2x Roche protease inhibitors at room temperature for 1 or 2 hours. Samples were then lysed by addition of 6x SDS loading dye (0.35 M Tris, pH 6.8, 30% glycerol, 10% SDS, 0.6 M DTT, and 0.012% bromophenol blue) to 1x. Samples (12 μL) were separated via 10% SDS-PAGE, and transferred to nitrocellulose using a Trans-Blot Turbo (BioRad) according to the manufacturer’s directions. Membranes were blocked in 5% milk in TBST (25 mM Tris, pH 7.5, 150 mM NaCl, and 0.1% Tween 20) at room temperature for 1 hour or at 4°C overnight. Blocking buffer was removed, and primary antibodies were added in 2% milk in TBST (αYlbL, 1:5000 or 1:8000; αCtpA, 1:5000; αRecA, 1:4000; αDnaN, 1:4000). Primary antibody incubation was performed at room temperature for 1 hour or overnight at 4°C. Primary antibodies were removed and membranes were washed three times with TBST for 5 minutes at room temperature. Secondary antibodies (Licor; 1:15000) were added in 2% milk in TBST and incubated at room temperature for 1 hour. Membranes were washed three times as above and imaged using the Li-COR Odyssey imaging system. All Western blot experiments were performed at least twice with independent samples. Molecular weight markers were used in the YlbL and CtpA blots. YlbL migrates to a location between the 30 and 40 kDa markers, consistent with its predicted molecular weight of 37.6 kDa. CtpA migrated to a location between the 40 and 80 kDa markers consistent with its molecular weight of 51.1 kDa.

For YneA Western blots, cell pellets, 10 mL OD_600_ = 1 for MMC recovery assay and 25 mL OD_600_ = 1 for over-expression, were re-suspended in 400 or 500 μL, respectively, of sonication buffer (50 mM Tris, pH 8.0, 10 mM EDTA, 20% glycerol, 2x Roche protease inhibitors, and 5 mM PMSF), and lysed via sonication. SDS loading dye was added to 2x and samples were incubated at 100°C for 7 minutes. Samples (10 μL) were separated using 16.5% Tris-Tricine-SDS-PAGE (BioRad) and transferred to a nitrocellulose membrane using a Trans-blot Turbo (BioRad) according to the manufacturer’s directions. All subsequent steps were performed as above with a 1:3000 primary antibody dilution.

### Mitomycin C recovery assay

An LB agar plate grown at 30°C overnight was washed with pre-warmed S7_50_ minimal media and used to inoculate a culture of S7_50_ minimal media at an OD_600_ = 0.1. The cultures were incubated at 30°C until an OD_600_ of about 0.2 (2–2.5 hours). MMC was added to 100 ng/mL and cultures were incubated at 30°C for 2 hours. Cells were pelleted via centrifugation (4,696 *g* for 7 minutes) and the media was removed. Cell pellets were washed in an equal volume of 1x PBS, pH 7.4, and pelleted again via centrifugation as above. Cell pellets were re-suspended in an equal volume of pre-warmed S7_50_ minimal media and incubated at 30°C for four hours. Samples for microscopy and Western blot analysis were taken after the two hour MMC treatment and at two and four hours following recovery, as indicated in the figures. The vehicle control samples were treated for 2 hours with an equivalent volume of the vehicle in which MMC was suspended (25% (v/v) DMSO).

### Microscopy

A 500 μL sample from the MMC recovery assay above was taken and FM4-64 was added to 2 μg/mL and incubated at room temperature for 5 minutes. Samples were then transferred to 1% agarose pads made of 1x Spizizen’s salts. Images were captured using an Olympus BX61 microscope.

### Cell length analysis

Cells were scored for cell length using the measuring tool in ImageJ software. For each image scored, all cells that were in focus were measured. The number of cells scored for each strain/condition is stated in the figures (n = cells measured). The histograms were generated using ggplot2 in R. All scoring was done using unadjusted images. Representative images shown in the figures were modified in ImageJ by subtracting the background (rolling ball radius method) and adjusting the brightness and contrast. Any adjustments made were applied to the entire image.

### Proteomics experimental details

Samples (5 mL OD_600_ = 1) were harvested from cultures grown as described in the MMC recovery assay section at 2 hours recovery via centrifugation: 4,696 *g* at room temperature for 10 minutes. Samples were washed twice with 500 μL 1x PBS, pH 7.4 and pelleted via centrifugation: 10,000 *g* at room temperature for 5 minutes. Samples were frozen in liquid nitrogen and stored at -80°C. Samples were submitted for mass-spectrometry analysis to MS Bioworks. Further sample processing and data analysis was performed by MS Bioworks as described in the supplemental methods. The raw data files are available upon request and the processed data tables are provided as supplemental tables (S4 and S5 Tables).

### YneA and lysozyme digestion assays

YneA digestion reactions were prepared as a 20 μL reaction in 20 mM Tris pH 7.5, 20 mM NaCl, and 20% glycerol containing 150 μM YneA, and 2 μM CtpA. Reactions were incubated at 30°C for the time indicated in the figure. Reactions were stopped by addition of 6x SDS-dye to 1x and incubating at 100°C for 5 minutes. Reaction products were separated via 16.5% Tris-Tricine SDS-PAGE. Proteins were detected by staining with coomassie blue. Lysozyme digestion assays were performed as for YneA using 2 mg/mL lysozyme and reactions were incubated at 30°C for 3 hours.

### Bacterial two-hybrid assays

Bacterial two hybrid assays were performed as previously described [47, 48, 63]. Briefly, T18 and T25 fusion plasmids were co-transformed into BTH101 cells and co-transformants were selected on LB agar + 100 μg/mL ampicillin + 50 μg/mL kanamycin at 37°C overnight. Cultures of LB + 100 μg/mL ampicillin + 50 μg/mL kanamycin were inoculated using several colonies and incubated at 37°C for 90 minutes. Cultures were diluted 250-fold and 4 μL were spotted on LB agar + 100 μg/mL ampicillin + 50 μg/mL kanamycin + 0.5 mM IPTG + 40 μg/mL X-gal and incubated at 30°C for 48 hours, then at room temperature for 24 hours. The brightness and contrast of the images were adjusted using Adobe Photoshop with changes applied to the entire image. All bacterial two-hybrid assays were performed at least twice.

## Supporting information

S1 Fig Tn-seq data analysis. **(A)** Sequencing read distributions for transposon (Tn) insertion locations containing greater than 0 reads for each replicate of the library samples (left) or the starter culture samples (right) from the MMC experiment. The y-axis is the frequency of Tn sites, and the x-axis is the log_10_ of sequencing reads. The dotted vertical line is drawn at log_10_(100). **(B)** Sequencing read distributions are plotted for the indicated samples as in panel A, except the replicates were summed prior to plotting. The library and starter cultures are the same in the Ctrl and MMC plots; the library and starter cultures are the same in the MMS and Phleo plots, which are shown twice in each case to allow direct comparison. The dotted vertical line is drawn at log_10_(350). **(C)** Relative fitness distributions are plotted for Tn insertions with more than 10 sequencing reads in the control samples. The y-axis is the frequency of Tn sites and the x-axis is the relative fitness. The dotted vertical line is drawn at 1.0. All three growth periods are plotted for the indicated experiments.
(TIF)

S2 Fig YlbL and CtpA catalytic residue identification. **(A)** The Lon protease domain of YlbL was aligned to LonA and LonB from *B*. *subtilis* and Lon from *E*. *coli*. The alignments show that YlbL contains the conserved catalytic dyad of Lon proteases consisting of serine 234 and lysine 279. **(B)** The S41 protease domain of CtpA was aligned to CtpB from *B*. *subtilis* and Prc from *E*. *coli*. The alignments showed that CtpA contains a conserved catalytic triad consisting of serine 297, lysine 322, and glutamine 326.
(TIF)

S3 Fig Disruption of *ylbK* results in a polar effect on *ylbL*. **(A)** Spot titer assay using the indicated genotypes and media. **(B)** Schematic of *ylbK* and *ylbL* loci, with the putative ribosome binding site, proposed to control *ylbL* translation, labeled in yellow. **(C)** Western blot analysis of cell lysates of the indicated genotypes using YlbL or DnaN antiserum.
(TIF)

S4 Fig YlbL and CtpA levels are not regulated by DNA damage. **(A)** MMC survival assay using strains with the indicated genotypes to test if MMC sensitivity is caused by cell death. The concentration of MMC used during a 30 minute incubation is listed on the x-axis, and the y-axis is the percent of cells surviving the treatment relative to the no treatment (0 ng/mL) condition. Each point is the average of three technical replicates from three individual experiments (n = 9), and the error bars represent the standard error of the mean. **(B)** Representative Western blot analysis of cell lysates throughout the MMC recovery assay using YlbL, CtpA, DnaN, or RecA antiserum. **(C)** Quantification of Western blot data plotted as a bar graph. The bars represent the average from three experiments (YlbL, CtpA, and DnaN) or two experiments (RecA), and the error bars are the standard deviation (YlbL and CtpA) or the range (RecA) of the measurements. The y-axis is the relative protein levels, which is the indicated protein level normalized to the loading control, DnaN, and the no treatment measurement.
(TIF)

S5 Fig YneA accumulates in protease mutants. **(A)** The averages of the normalized spectral counts are plotted as histograms for WT (blue) and Δ*ylbL*, Δ*ctpA* double mutant (DM; red). The y-axis is the count and the x-axis is the log_10_(normalized spectral counts for the average of three replicates). **(B)** The distribution of the test statistic (WT average–DM average) is plotted as a histogram. **(C)** A principle component analysis was performed using the normalized spectral counts from WT (blue) and DM (red) samples using the “prcomp” function in R. The first two coordinates are plotted as the x- and y-axes, respectively. **(D & E)** The average relative protein levels (WT/DM) from the proteomics dataset are plotted for the indicated proteins, and the error bars represent the standard deviation. The inset in panel E shows a closer look around one for clarity.
(TIF)

S6 Fig *yneA* is required for DNA damage sensitivity and cell elongation phenotypes. **(A)** Strains with the indicated genotypes (plates at left) were struck onto the indicated media (column labels) and incubated at 30°C overnight. Deletion of *yneA* suppresses the Δ*ylbL*, Δ*ctpA* double mutant (DM) MMC sensitivity phenotype. **(B)** Representative micrographs of cells stained with FM4-64 from the indicated genotypes at the indicated time points in the MMC recovery assay. The scale bar is 5 μm.
(TIF)

S7 Fig Purified YlbL lacking its N-terminal transmembrane shows no protease activity *in vitro*. **(A)** Protease assay using fluorescently labeled casein. YlbL, YlbL-S234A, CtpA, and CtpA-S297A, all lacking their N-terminal transmembrane domains were incubated with casein. The casein was fluorescently labeled such that the signal was quenched until digested by a protease. **(B)** YneA digestion assay. YlbL and CtpA were incubated with increasing concentrations of YneA. The first lane is a molecular weight marker (M). **(C)** Lysozyme digestion assay. YlbL was incubated with lysozyme.
(TIF)

S1 Table Tn-seq data collected. OD_600_ measurements, incubation times, viable cell counts, growth rates estimated based on viable cell counts, number of generations estimated based on viable cell counts and incubation times, sequencing sample IDs, sequencing reads, reads mapped, and the number of Tn insertions with more than 10 reads for each sample are presented.
(XLSX)

S2 Table Tn-seq relative fitness lists. The relative fitness values for each gene with sufficient data in all three growth periods for all three Tn-seq experiments are presented along with the adjusted p-value (BH method; see STAR methods). The gene names or locus tags are listed, and intergenic regions are annotated as “ig” with a number.
(XLSX)

S3 Table Tn-seq list overlaps. **(First tab)** The genes with the lowest relative fitness with an adjusted p-value less than 0.01 are listed for each experiment. **(Second tab)** The genes overlapping in all three Tn-seq experiments as well as genes overlapping in pairwise comparisons of the three Tn-seq experiments are presented.
(XLSX)

S4 Table Extended proteomics data set. The spectral counts for all proteins detected and the statistical analysis of differences between WT and double mutant cell extracts.
(XLSX)

S5 Table Proteomics data set. The proteomics data for the 183 proteins identified as having significantly different levels (p-value < 0.05) in the protease double mutant relative to the control are presented.
(XLSX)

S6 Table Oligonucleotides, plasmids, and strains used in this study. (XLSX)

S7 Table Numerical data underlying graphs. The numerical data underlying all graphs is presented.
(XLSX)

S1 Text Supplemental results and methods. (PDF)
