# Supplementary figures and images for: Discovery of a dual protease mechanism that promotes DNA damage checkpoint recovery
Source: PLoS Genet. 2018 Jul 6;14(7):e1007512. doi: 10.1371/journal.pgen.1007512 (PMC6051672; doi:10.1371/journal.pgen.1007512)

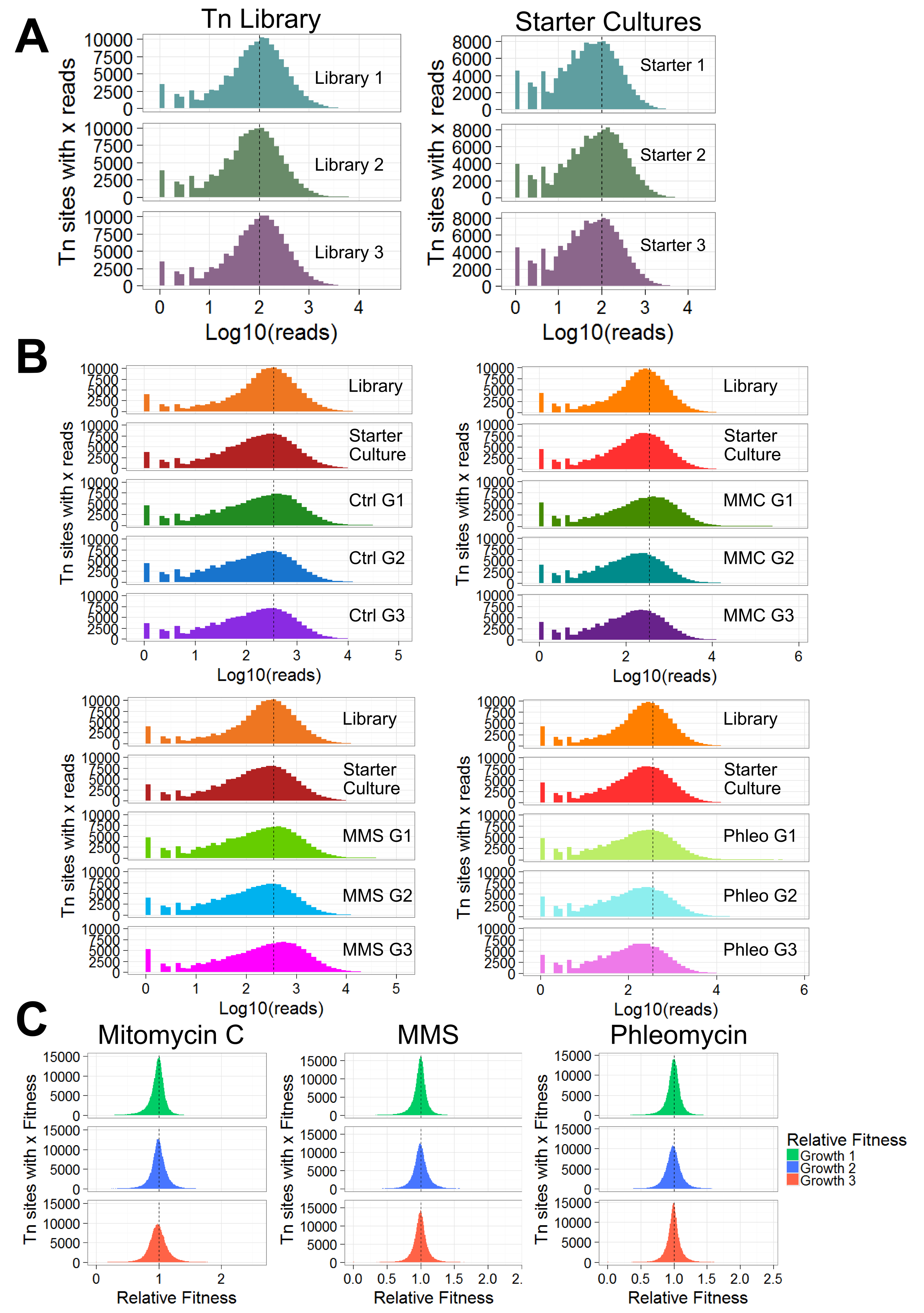

Supplement: S1 Fig — (A) Sequencing read distributions for transposon (Tn) insertion locations containing greater than 0 reads for each replicate of the library samples (left) or the starter culture samples (right) from the MMC experiment. The y-axis is the frequency of Tn sites, and the x-axis is the log10 of sequencing reads. The dotted vertical line is drawn at log10(100). (B) Sequencing read distributions are plotted for the indicated samples as in panel A, except the replicates were summed prior to plotting. The library and starter cultures are the same in the Ctrl and MMC plots; the library and starter cultures are the same in the MMS and Phleo plots, which are shown twice in each case to allow direct comparison. The dotted vertical line is drawn at log10(350). (C) Relative fitness distributions are plotted for Tn insertions with more than 10 sequencing reads in the control samples. The y-axis is the frequency of Tn sites and the x-axis is the relative fitness. The dotted vertical line is drawn at 1.0. All three growth periods are plotted for the indicated experiments. (TIF) [file pgen.1007512.s001.tif]

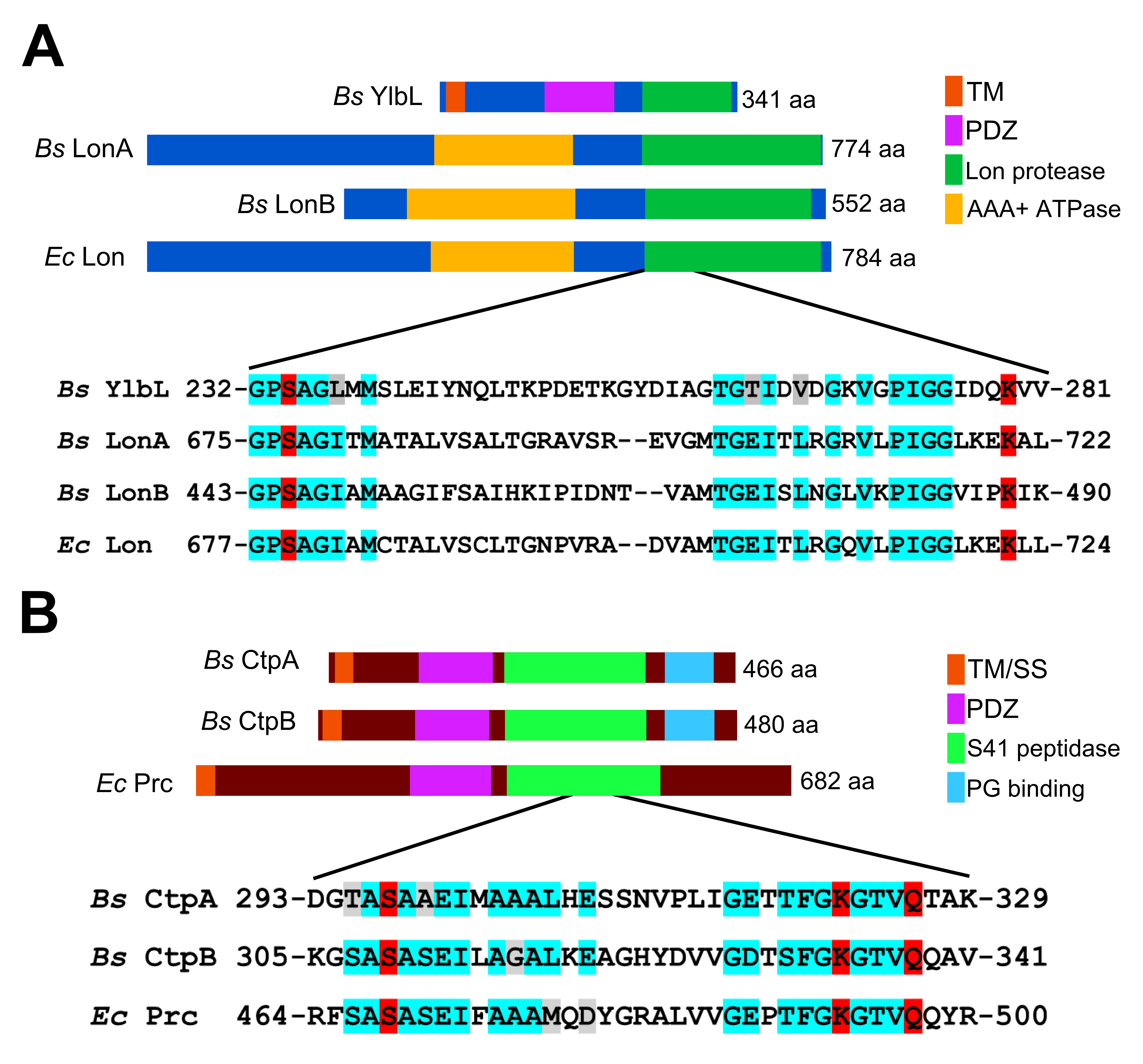

Supplement: S2 Fig — (A) The Lon protease domain of YlbL was aligned to LonA and LonB from B. subtilis and Lon from E. coli. The alignments show that YlbL contains the conserved catalytic dyad of Lon proteases consisting of serine 234 and lysine 279. (B) The S41 protease domain of CtpA was aligned to CtpB from B. subtilis and Prc from E. coli. The alignments showed that CtpA contains a conserved catalytic triad consisting of serine 297, lysine 322, and glutamine 326. (TIF) [file pgen.1007512.s002.tif]

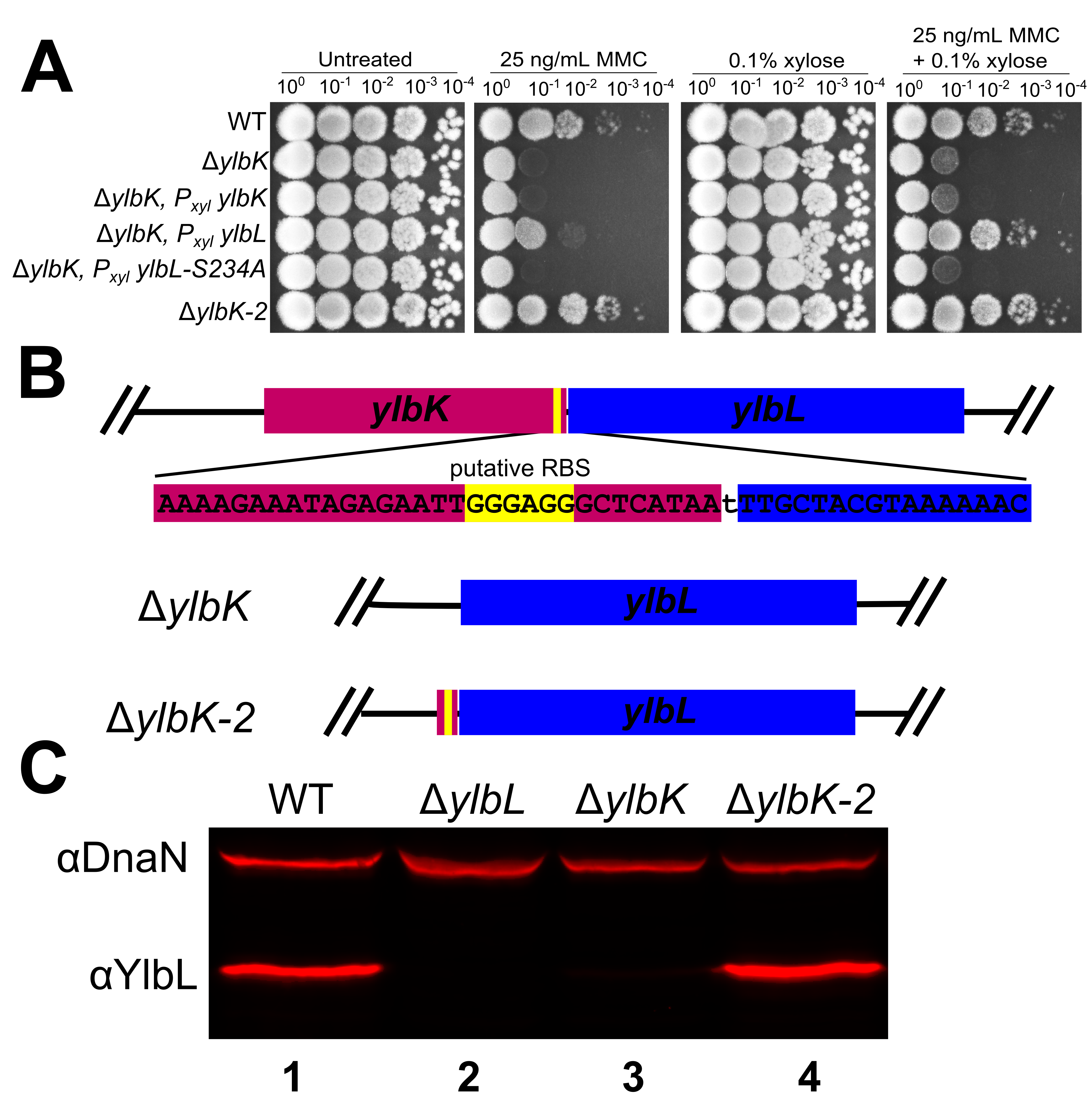

Supplement: S3 Fig — (A) Spot titer assay using the indicated genotypes and media. (B) Schematic of ylbK and ylbL loci, with the putative ribosome binding site, proposed to control ylbL translation, labeled in yellow. (C) Western blot analysis of cell lysates of the indicated genotypes using YlbL or DnaN antiserum. (TIF) [file pgen.1007512.s003.tif]

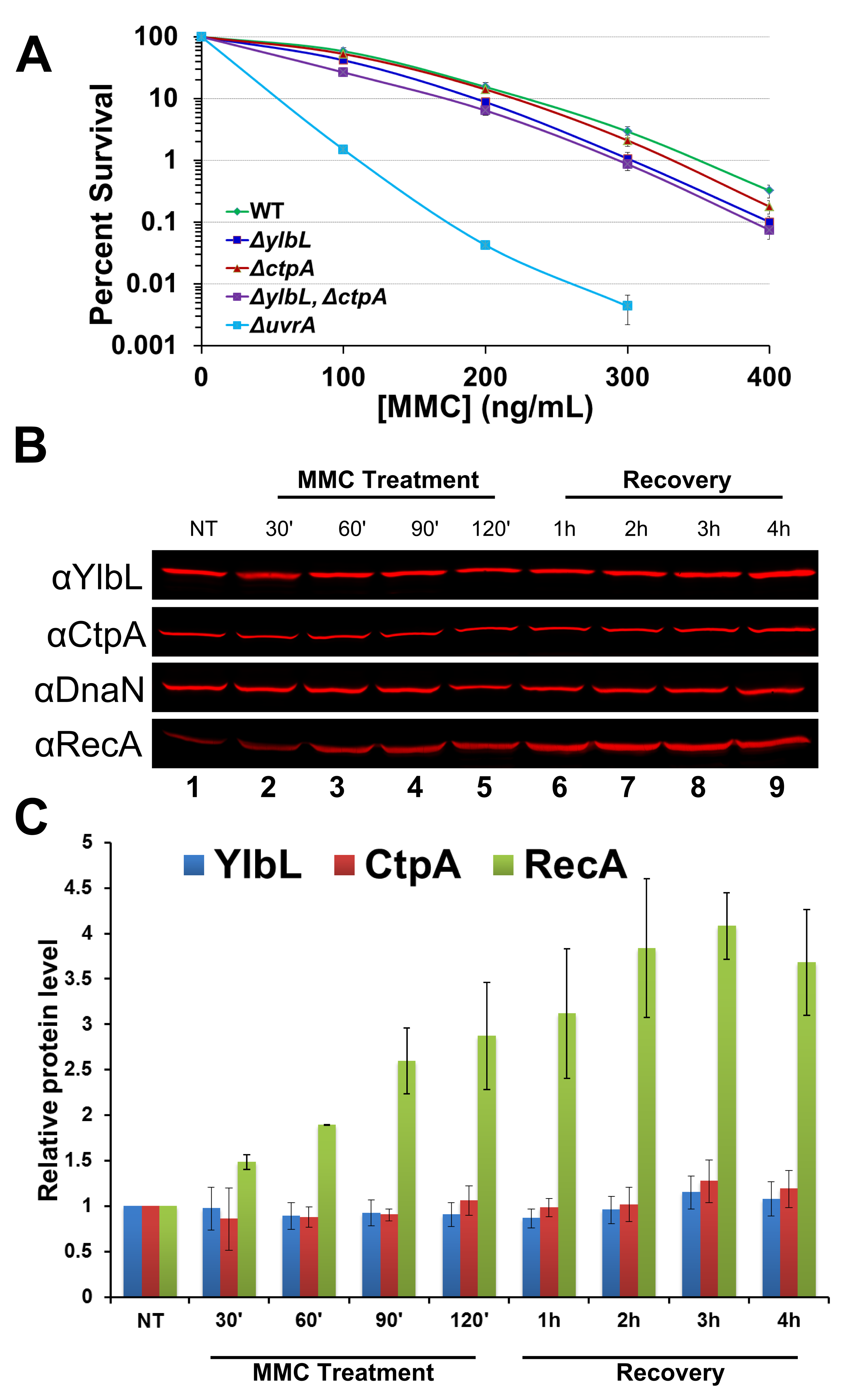

Supplement: S4 Fig — (A) MMC survival assay using strains with the indicated genotypes to test if MMC sensitivity is caused by cell death. The concentration of MMC used during a 30 minute incubation is listed on the x-axis, and the y-axis is the percent of cells surviving the treatment relative to the no treatment (0 ng/mL) condition. Each point is the average of three technical replicates from three individual experiments (n = 9), and the error bars represent the standard error of the mean. (B) Representative Western blot analysis of cell lysates throughout the MMC recovery assay using YlbL, CtpA, DnaN, or RecA antiserum. (C) Quantification of Western blot data plotted as a bar graph. The bars represent the average from three experiments (YlbL, CtpA, and DnaN) or two experiments (RecA), and the error bars are the standard deviation (YlbL and CtpA) or the range (RecA) of the measurements. The y-axis is the relative protein levels, which is the indicated protein level normalized to the loading control, DnaN, and the no treatment measurement. (TIF) [file pgen.1007512.s004.tif]

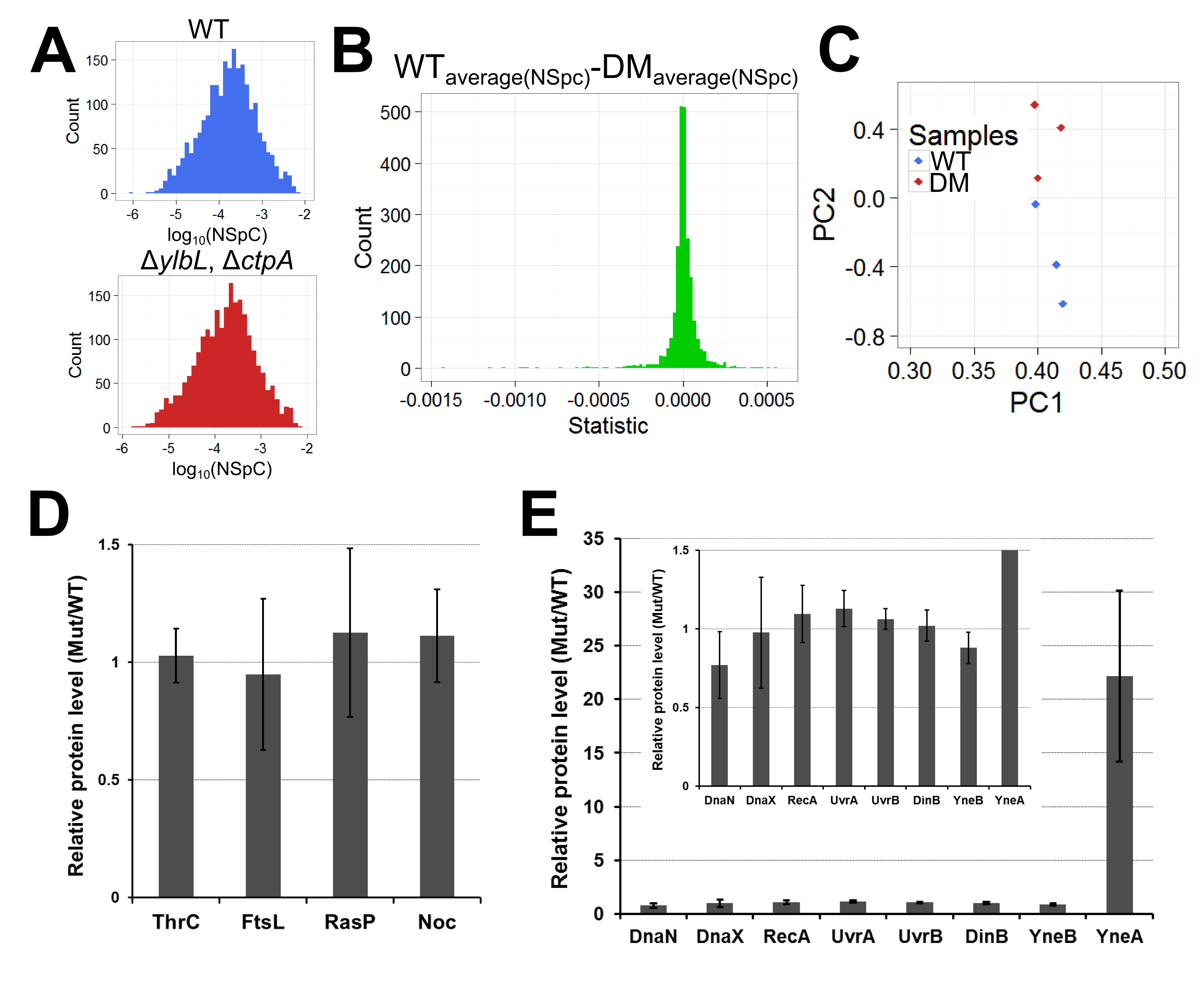

Supplement: S5 Fig — (A) The averages of the normalized spectral counts are plotted as histograms for WT (blue) and ΔylbL, ΔctpA double mutant (DM; red). The y-axis is the count and the x-axis is the log10(normalized spectral counts for the average of three replicates). (B) The distribution of the test statistic (WT average–DM average) is plotted as a histogram. (C) A principle component analysis was performed using the normalized spectral counts from WT (blue) and DM (red) samples using the “prcomp” function in R. The first two coordinates are plotted as the x- and y-axes, respectively. (D & E) The average relative protein levels (WT/DM) from the proteomics dataset are plotted for the indicated proteins, and the error bars represent the standard deviation. The inset in panel E shows a closer look around one for clarity. (TIF) [file pgen.1007512.s005.tif]

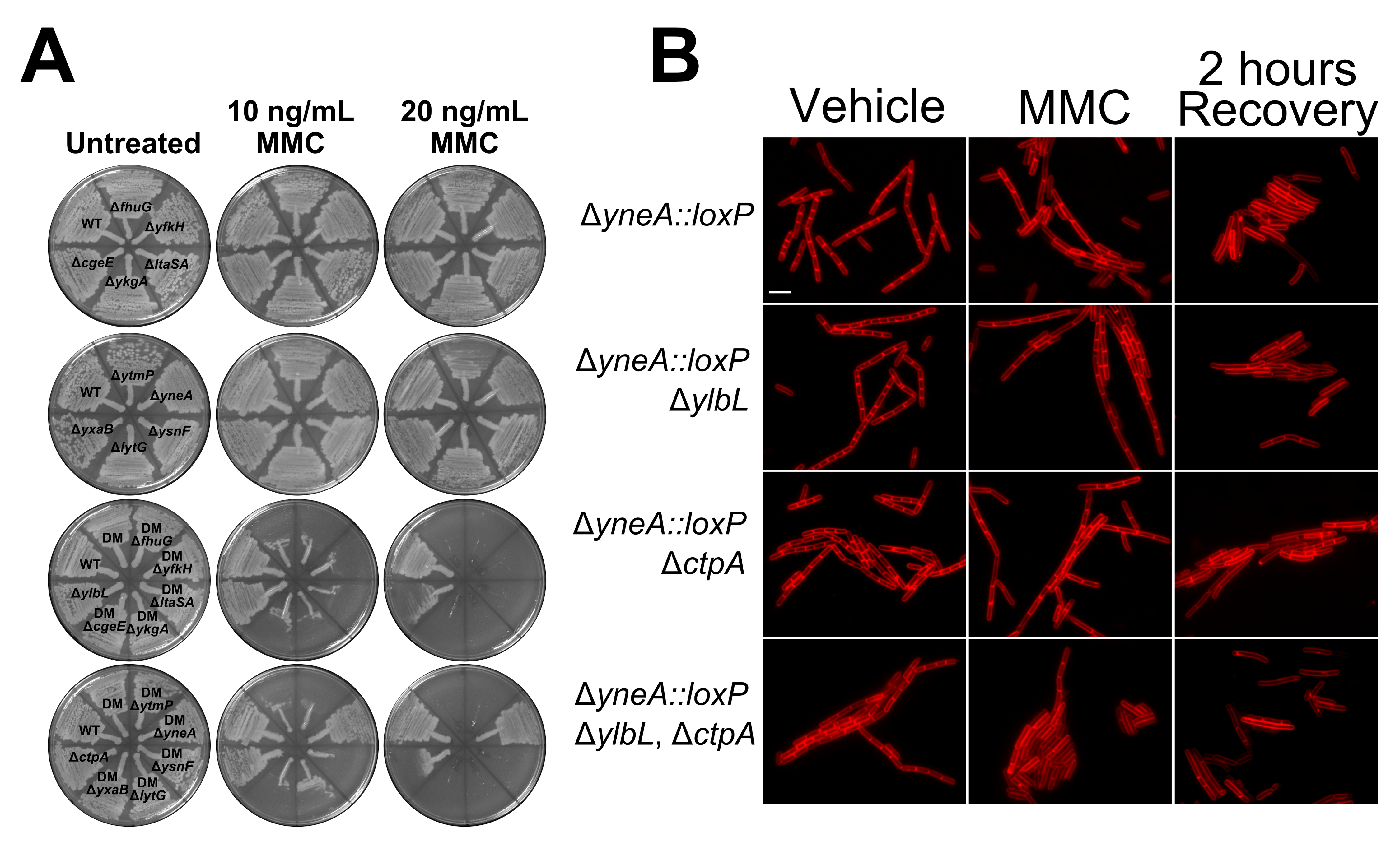

Supplement: S6 Fig — (A) Strains with the indicated genotypes (plates at left) were struck onto the indicated media (column labels) and incubated at 30°C overnight. Deletion of yneA suppresses the ΔylbL, ΔctpA double mutant (DM) MMC sensitivity phenotype. (B) Representative micrographs of cells stained with FM4-64 from the indicated genotypes at the indicated time points in the MMC recovery assay. The scale bar is 5 μm. (TIF) [file pgen.1007512.s006.tif]

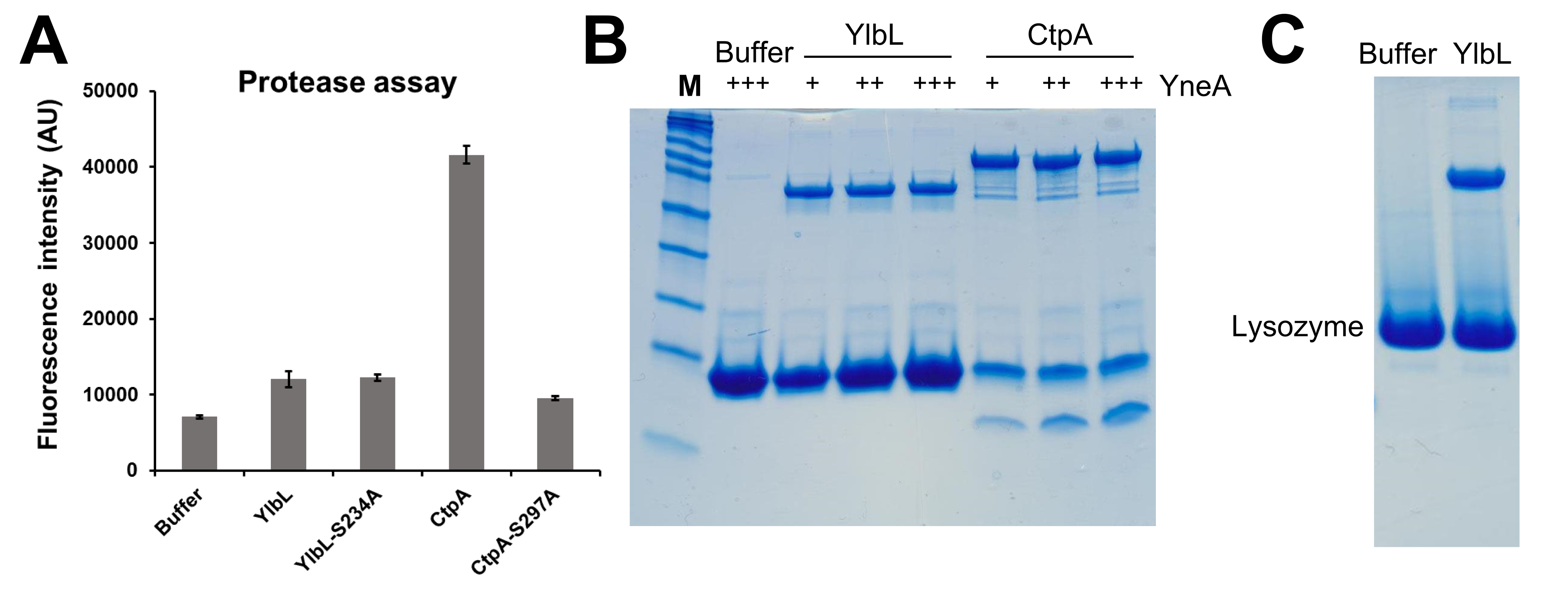

Supplement: S7 Fig — (A) Protease assay using fluorescently labeled casein. YlbL, YlbL-S234A, CtpA, and CtpA-S297A, all lacking their N-terminal transmembrane domains were incubated with casein. The casein was fluorescently labeled such that the signal was quenched until digested by a protease. (B) YneA digestion assay. YlbL and CtpA were incubated with increasing concentrations of YneA. The first lane is a molecular weight marker (M). (C) Lysozyme digestion assay. YlbL was incubated with lysozyme. (TIF) [file pgen.1007512.s007.tif]
