# Supplementary material for: Discovery of a dual protease mechanism that promotes DNA damage checkpoint recovery
Source: PLoS Genet. 2018 Jul 6;14(7):e1007512. doi: 10.1371/journal.pgen.1007512 (PMC6051672; doi:10.1371/journal.pgen.1007512)
Supplement: S1 Text — (PDF) [file pgen.1007512.s015.pdf]

Supplemental Text

Discovery of a dual protease mechanism that promotes DNA damage checkpoint recovery

Peter E. Burby, Zackary W. Simmons, Jeremy W. Schroeder, Lyle A. Simmons*

Department of Molecular, Cellular, and Developmental Biology, University of Michigan, Ann Arbor, MI 48109.

*Correspondence: lasimm@umich.edu

Supplemental Results

ylbK* disruption results in a polar effect on *ylbL

We noticed that *ylbK*, the gene upstream of *ylbL*, had a phenotype similar to *ylbL* in the Tn-seq experiments (Table S2 & S3). Therefore, we tested whether *ylbKL* functioned together in the DNA damage response. Deletion of *ylbK* resulted in sensitivity to MMC (Fig S3A). Ectopic expression of *ylbK* failed to complement the Δ*ylbK* phenotype (Fig S3A). Given that *ylbK* is upstream of *ylbL* we attempted to complement the Δ*ylbK* phenotype using *ylbL* and found that sensitivity to MMC was rescued (Fig S3A). Closer examination of the *ylbKL* locus revealed that a putative ribosome binding site (RBS) for *ylbL* translation was present within the 3′ end of *ylbK* (Fig S3B). Thus, a second deletion of *ylbK* was made (Δ*ylbK-2*) which included deletion of the codons for all but the first 3 and the last 14 amino acids, leaving the RBS for *ylbL* intact (Fig S3B). This deletion was not sensitive to MMC (Fig S3A). Western blotting revealed that the initial Δ*ylbK* strain did not express YlbL, whereas Δ*ylbK-2* did (Fig S3C). We conclude that disruption of *ylbK* results in a polar effect on *ylbL*, indicating that YlbL functions independently of YlbK.

In order to better understand the prevalence of false positives in Tn-seq experiments we attempted to validate the MMC phenotypes of the forty genes with the lowest relative fitness values in the second growth period of the experiment. Intriguingly, we found that seven additional genes, *queA*, *ylmG*, *lgt*, *ylmE*, *sdaAB*, *cymR*, and *ywrC*, resulted in no sensitivity to MMC when deleted (Table 1). We also found that the genomic loci of *queA*, *cymR*, *ylmG*, *ylmE*, and *sdaAB*, were proximal to genes with validated phenotypes (Table 1). The other two genes, *ywrC* and *lgt* have less obvious explanations. For *lgt* it is possible that the polar effect is on the

upstream gene *hprK*, which codes for the kinase HprK that phosphorylates Crh (1). Deletion of *crh* resulted in sensitivity to MMC (Table 1), but we did not detect *hprK* in our Tn-seq experiments. Finally, *ywrC* does not have a clear explanation. We could not identify validated mutant phenotypes or essential genes proximal to *ywrC*. It is possible that the transposon resulted in increased expression of neighboring genes that resulted in sensitivity to MMC, and that increased expression is not duplicated in the deletion mutant, though other explanations exist. Taken together, our results underscore the importance of validating results from forward genetic screens.

Cell wall metabolism genes are sensitive to DNA damage

Our forward genetic screens identified several cell wall metabolism genes as being sensitive to DNA damaging agents, including *walH*, *yycI*, *walJ*, *ponA*, and *brcC* (Table 1 and Table S2). We validated that deletion mutants were indeed sensitive to MMC (Table 1). These genes have not previously been implicated in the DNA damage response, though it is possible that they function in regulating cell division. Specifically, the genes *walH* and *yycI* are negative regulators of the essential two-component system WalRK (2-5). A recent publication provided evidence that the WalRK system interacts with components of the divisome (6). Further, a study of *walJ* found that WalJ likely coordinates cell division with DNA replication (7). As a result, it is tempting to speculate that WalRK and the associated WalHIJ represent one of the connections between DNA replication, the DNA damage response, cell wall metabolism, and cell division.

Supplemental Materials and Methods

Transposon insertion mutant library construction

Extraction of PY79 chromosomal DNA

Cell pellets (10 mL OD_{600} = 1 equivalent) from stationary phase cultures were re-suspended in lysis buffer (50 mM Tris, pH 8.0, 10 mM EDTA, pH 8.0, 1% (v/v) Triton X-100, 0.5 mg/mL RNase A, 1 mg/mL lysozyme) and incubated at 37°C for 30 minutes. Proteins were digested by addition of 40 µL 10 mg/mL proteinase K (dissolved in TE buffer plus 10% glycerol) and 30 µL of 10% SDS and incubated at 55°C for 30 minutes. 600 µL of PB buffer (5 M guanidine-HCl, and 30% (v/v) isopropanol) were added, mixed well by pipetting and added directly to a silica spin column (Epoch life sciences) and centrifuged at 12,000 *g* for 1 minute at room temperature. The column was washed with 500 µL PB buffer, then 750 µL PE buffer (10 mM Tris, pH 7.5, and 80% (v/v) ethanol), centrifuging as above to remove buffer. The column was dried by centrifugation as above. Chromosomal DNA was eluted by adding 100 µL ultra-pure water and centrifugation as above.

Purification of Himar1-C9 transposase

Himar1-C9 was purified as described previously (8). *E. coli* TB1 cells with plasmid pMalC9 (Strain PEB234) were struck out on LB + 100 µg/mL ampicillin and incubated at 37°C overnight. An overnight starter culture was grown in LB + 100 µg/mL ampicillin at 37°C. The starter culture was diluted 1:100 and incubated at 37°C until OD_{600} = 0.5 and Himar1-C9 was induced by addition of IPTG to a final concentration of 0.3 mM. The culture was incubated for 2 hours at 37°C. Cells were collected via centrifugation: 5,000 *g* for 20 minutes at 4°C. Cell pellets were re-suspended in 20 mL ice-cold column buffer (CB; 20 mM Tris, pH 7.5, 200 mM NaCl, 1

mM EDTA, and 1x Roche protease inhibitors). Cells were lysed via French press, and the lysate was clarified via centrifugation: 18,000 rpm (Sorvall SS-34 rotor) for 30 minutes at 4°C. The lysate was loaded onto 1 mL amylose resin (NEB) pre-equilibrated with CB, and placed on a rotator at 4°C for 1 hour. Resin was collected via centrifugation: 3,000 *g* for 10 minutes at 4°C. The supernatant was removed and the resin was washed with 4 volumes wash buffer (20 mM Tris, pH 7.5, 200 mM NaCl, 1 mM EDTA, 2 mM DTT, and 10% (v/v) glycerol) five times by re-suspending the resin, then collecting via centrifugation and aspirating the wash buffer. The column was eluted by adding 0.8 volume of elution buffer (20 mM Tris-HCl, pH 7.5, 200 mM NaCl, 1 mM EDTA, 2 mM DTT, 10% (v/v) glycerol, and 10 mM maltose) and incubating on ice for 5 minutes and agitating frequently. The protein was analyzed via SDS-PAGE and concentration was determined using a Bradford assay. The protein was aliquoted, frozen in liquid nitrogen and stored at -80°C.

Transposition reaction

A transposon insertion library was constructed *in vitro* as described with minor modifications (9). A 50 μL transposition was prepared: 3 μg chromosomal DNA, 1 μg PCR product mariner transposon (The mariner transposon was PCR amplified from pCJ41 using primers oPEB368/369 and Q5 DNA polymerase (NEB)), 10 μL 5x Buffer A (102.5 mM HEPES pH 7.9, 47.5% glycerol, 467.5 mM NaCl, 47.5 mM $MgCl_2$, 1.19 mg/mL BSA, 9.5 mM DTT), 100 nM Himar1-C9. Reactions were incubated at 30 °C for 16 hours. DNA was precipitated via addition of 0.1 volume sodium acetate, pH 5.2 and 2.5 volume 100% ice-cold ethanol. DNA was collected via centrifugation: 16,000g for 20 minutes at 4°C. DNA pellet was washed with 5 volumes (relative to reaction volume) ice-cold 70% ethanol and pelleted again via centrifugation: 16,000g for 10 minutes at 4°C. The ethanol wash was aspirated and the DNA pellet was dried at

room temperature. The transposon junctions were repaired by re-suspending the DNA pellet in the following 50 µL reaction: 1x T_4 DNA ligase buffer (Lucigen), 1 mg/mL BSA (NEB), 0.5 mM dNTPs, 1 mM ATP, 50 mM NaCl, 6 units T_4 DNA polymerase exo- (Lucigen), and 480 units T_4 DNA ligase (Lucigen), and incubating at room temperature for 2 hours. The reactions were mixed by pipetting every 30 minutes over the 2 hour incubation. The reactions were moved to 16°C for 16 hours, and then stored at 4°C until used to transform PY79.

Transformation of PY79

PY79 was struck out on LB agar and incubated at 37°C overnight. A single colony was used to inoculate a 2 mL LM culture (LB + 3 mM $MgSO_4$) in a 14 mL round bottom culture tube. The culture was incubated at 37°C on a rolling rack until OD_{600} of about 1.5. Then, 40 µL of the LM culture was transferred to 1.2 mL pre-warmed MD media (1x PC buffer (107 g/L K_2HPO4, 60 g/L KH_2PO_4, 11.8 g/L trisodium citrate dihydrate), 2% glucose, 50 µg/mL phenylalanine, 50 µg/mL tryptophan, 11 µg/mL ferric ammonium citrate, 2.5 mg/mL potassium aspartate, 3 mM $MgSO_4$) and incubated on a rolling rack at 37°C for 6 hours. To each 1.2 mL competent cell culture, 15 µL of the transposase reaction were added, and the cultures were incubated on a rolling rack at 37°C for an additional 1.5 hours. Transformations were plated on LB agar + 100 µg/mL spectinomycin (200 µL per 100 cm plate, and 124 plates in total), and incubated at 37°C overnight. The Library consisted of approximately 900,000 transformants, which were pooled in 1x $S7_{50}$ salts + 15% glycerol with a resulting OD_{600} of approximately 37.0. The library was distributed into 1 mL aliquots, frozen in liquid nitrogen, and stored at -80°C.

Tn-seq experimental details

Tn-seq experiments were designed with multiple growth periods similar to a prior description (10). The experiment was performed using triplicate samples for each condition, which originated from three aliquots of the transposon insertion library. The experiment was initiated by thawing three aliquots of the transposon insertion library in a beaker of water at 37°C. Each aliquot of the thawed library was used to inoculate 50 mL starter cultures in 500 mL beakers. For the MMC experiment 270 μL were used to inoculate, and 500 μL were used for the MMS and phleomycin experiment. The three starter cultures were incubated with shaking (200 rpm) at 30°C until OD_{600} of about 0.8. Starter cultures were used to inoculate paired 25 mL cultures in 250 mL flasks at an OD_{600} = 0.05 for control or treatment. For the MMC experiment, MMC was added to a final concentration of 15 ng/mL and an equal volume of the vehicle in which MMC was dissolved (25% v/v DMSO) was added to the control flasks. For MMS, a final concentration of 50 μg/mL was used, and a final concentration of 25 ng/mL was used for phleomycin. An equal volume of water was used for the vehicle control in the MMS and phleomycin experiment. The paired cultures were incubated with shaking (200 rpm) at 30°C until OD_{600} of about 1.5 (growth period 1). Then the cultures were back diluted into fresh control or treatment media at an OD_{600} = 0.05, and incubated with shaking (200 rpm) at 30°C until OD_{600} of about 1.5 (growth period 2). The cultures were back diluted as above one more time and grown as above (growth period 3). At all steps of the experiment three samples of OD_{600} = 10 were saved as cell pellets, and each sample was serially diluted and plated for viable cells in triplicate to estimate the number of cells at the start and end of the growth periods (Table S1), which were used in the fitness calculations.

Tn-seq sequencing library construction

Sequencing libraries were prepared similar to previous reports (9, 11), with some modifications in adaptor sequences to increase compatibility with standard Hi-seq reagents. Genomic DNA was extracted from each sample as described in "Extraction of PY79 genomic DNA." A 200 µL restriction digest using MmeI was assembled for each sample as follows: 6 µg gDNA, 1x Cutsmart buffer (NEB), 64 µM SAM (NEB), 12 units MmeI (NEB). Reactions were incubated at 37°C for 4 hours. CIP (20 units) was added and the reactions were incubated at 37°C for 1 hour. MmeI was heat inactivated by incubating at 65°C for 30 minutes, and the digested genomic DNA was extracted by addition of 600 µL of PB buffer and binding to a silica spin-column. The column was washed with 500 µL PB, then 750 µL PE buffer, and then eluted with 65 µL ultra-pure water. Adaptors (oPEB312/313) were annealed in 1x annealing buffer (10 mM Tris, pH 8.0, 100 mM NaCl, 10 µM EDTA) at a concentration of 25 mM each, by boiling at 100°C for 5 minutes, followed by transferring to a beaker of 100°C water which was allowed to cool slowly to room temperature. The adaptors were ligated to the digested genomic DNA in two 40 µL reactions, each containing 30 µL of the eluate from above, 1x T_4 DNA ligase buffer (NEB), 2.5 µM annealed adaptors, and 800 units T_4 DNA ligase (NEB). Reactions were left at room temperature for 30 minutes, and then transferred to 16°C for 16 hours. The resulting reactions were pooled for each sample, and the DNA was extracted via spin-column as above, but the PB wash was excluded, and the sample was eluted with 100 µL ultra-pure water.

Transposon/genomic DNA junctions were PCR amplified in two 50 µL reactions each containing 4 µL of eluate from the previous step, 1x Q5 DNA polymerase buffer (NEB), 200 µM dNTPs, 400 nM forward primer (oPEB314), 400 nM reverse primer with multiplexing barcode (one of oPEB315-326), and 1 unit of Q5 DNA polymerase (NEB). The reactions were cycled as follows:

1) 98°C for 3 minutes; 2) 98°C for 30 seconds; 3) 55°C for 30 seconds; 4) 72°C for 30 seconds; 5) repeat steps 2-4 for 18 cycles; 6) 72°C for 2 minutes; 7) hold at 4°C. The reactions were separated on a 2.5% agarose gel and the reaction product at approximately 160 bp was excised. The DNA was extracted by dissolving the gel slice in 400 μL QG buffer (5.5 M guanidine thiocyanate and 20 mM Tris, pH 6.6) by heating at 65°C, and then 250 μL of isopropanol were added and the mixture was added to a silica spin-column. The column was washed with PB and PE buffer as above, and eluted with 120 μL ultra-pure water. The eluate was submitted for sequencing using a Hi-seq 2500 instrument on high output mode with v3 (MMC experiment) or v4 reagents (MMS and phleomycin experiment).

Tn-seq data analysis

The 50 bp sequencing reads were trimmed to 43 bp using Fastx Trimmer, because the sequencing reaction went outside of the inverted repeat, and those seven base pairs could not be aligned to our reference database. The trimmed reads were aligned to a reference database in which every TA site found in the PY79 genome was placed adjacent to the transposon sequence (reference database fasta file and sequencing data accession number GSE109366) using bwa (12). The reads were imported into the R statistical software package RStudio (RStudio 13) for further analysis. Each transposon insertion site was provided a coverage value where each read was equal to a coverage of one. Each transposon insertion site was indexed to its position in the genome and the gene or intergenic region in which it resides. Fitness was calculated for each insertion for the control and the treatment using the equation:

$$W = \frac{\ln \frac{(N_f \cdot F_f)}{N_0 \cdot F_0}}{\ln \frac{N_f(1 - F_f)}{N_0(1 - F_0)}}$$

where N_0 and N_f are the number of bacteria at the start and end of the growth period, respectively, and F_0 and F_f are the transposon frequency in the population as measured by Illumina sequencing (insertion coverage divided by total reads in the sample), respectively (11; also see Fig 1C). The ratio of treatment to control was calculated and defined as the relative fitness (see Fig 1C). For each gene (or intergenic region), the insertions containing less than 10 reads were removed and any gene without at least 12 insertions from the combined triplicate data were also removed, thus requiring that each gene have at least 4 insertions in each replicate to be included in the analysis. Each gene's average relative fitness was then calculated by determining the mean relative fitness of all insertion sites within each gene. We trimmed insertions in our fitness calculation that were in the upper or lower five percent of the data for that gene, reasoning that not all insertions will be a true representation of a null allele. A t-test was used to determine if the gene relative fitness differed significantly from one. P-values were adjusted for multiple comparisons using the method of Benjamini and Hochberg (14).

Proteomics experimental details

Ms Bioworks processed samples as described below. Submitted samples were washed three times with PBS. The washed pellets were suspended in modified RIPA buffer (2% SDS, 150 mM NaCl, 50 mM Tris HCl pH 8) and lysed using mechanical disruption in a Next Advance Bullet Blender using 1.0mm silica beads, setting 8 for 3 minutes. The lysate was centrifuged at 10,000 g for 10 minutes. Protein concentrations were determined by Qubit fluorometry. 20 µg of each sample was processed by SDS-PAGE using a 10% Bis-Tris NuPAGE gel (Invitrogen) with the MES buffer system; the gel was electrophoresed approximately 5 cm. The mobility region was excised into 20 equal sized segments for further processing by in-gel digestion. In-gel digestion was performed on each submitted sample using a robot (ProGest, DigiLab) with the

following protocol: 1) Washed with 25mM ammonium bicarbonate followed by acetonitrile. 2) Reduced with 10mM dithiothreitol at 60°C followed by alkylation with 50mM iodoacetamide at room temperature. 3) Digested with trypsin (Promega) at 37°C for 4h. 4) Quenched with formic acid and the supernatant was analyzed directly without further processing.

Each gel digest was analyzed by nano LC-MS/MS with a Waters NanoAcquity HPLC system interfaced to a ThermoFisher Q Exactive. Peptides were loaded on a trapping column and eluted over a 75µm analytical column at 350 nL/min; both columns were packed with Luna C18 resin (Phenomenex). The mass spectrometer was operated in data-dependent mode, with the Orbitrap operating at 60,000 FWHM and 17,500 FWHM for MS and MS/MS, respectively. The fifteen most abundant ions were selected for MS/MS.

Proteomics data analysis

Data were processed as described by MS Bioworks. Data were searched using a local copy of Mascot with the following parameters: Enzyme: Trypsin/P; Database: UniProt *Bacillus subtilis* (concatenated forward and reverse plus common potential contaminants); Fixed modification: Carbamidomethyl (C); Variable modifications: Oxidation (M), Acetyl (N-term), Pyro-Glu (N-term Q), Deamidation (N/Q); Mass values: Monoisotopic; Peptide Mass Tolerance: 10 ppm; Fragment Mass Tolerance: 0.02Da; Max Missed Cleavages: 2. Mascot DAT files were parsed into Scaffold (Proteome Sciences) for validation, filtering and to create a non-redundant list per sample. Data were filtered using at 1% protein and peptide FDR and requiring at least two unique peptides per protein. A student's t-test was performed to determine if wild-type and the double mutant levels were significantly different ($\alpha = 0.05$).

Protein purification

YlbL

YlbL for antibody production was purified as follows. 10xHis-Smt3-YlbL (a.a 36-341) was expressed from plasmid pPB157 in *E. coli* NiCo21 cells (NEBC2529H). One liter LB + 50 μg/mL kanamycin cultures were inoculated with an overnight culture (1:100) and grown at 37°C with shaking (200 rpm) until OD_{600} of about 0.5. Cultures were iced briefly and IPTG was added to 0.5 mM and incubated at 20°C with shaking overnight. Cells were harvested via centrifugation: 4,000 *g* for 20 minutes at 4°C. Cell pellets were re-suspended in lysis buffer (50 mM potassium phosphate pH 8.0, 300 mM NaCl, 5% (v/v) glycerol, and 20 mM imidazole). Cells were lysed via sonication and lysates were cleared via centrifugation: 18,000 rpm (Sorvall SS-34 rotor) for 45 minutes at 4°C. The supernatant was removed and incubated with Ni^{2+}-NTA-agarose (Qiagen) for 1 hour at 4°C. The lysate/bead slurry was loaded onto a gravity flow column and the beads were allowed to settle for 5-10 minutes. The lysate was collected as the flow-through. The column was washed with 50 column volumes wash buffer (50 mM potassium phosphate pH 8.0, 300 mM NaCl, 5% (v/v) glycerol, and 30 mM imidazole). YlbL was eluted from the column via digestion with 6xHis-Ulp1 in digestion buffer (50 mM potassium phosphate pH 8.0, 150 mM NaCl, 5% (v/v) glycerol, 1 mM DTT, and 10 mM imidazole) on a rotator at room temperature for 2 hours, yielding untagged YlbL (a.a. 36-341). The digestion buffer was collected as the flow-through and concentrated to approximately 5 mL using a 10 kDa Amicon centrifugal filter. YlbL was then loaded onto a HiLoad superdex 200-PG 16/60 column pre-equilibrated with SEC buffer (50 mM potassium phosphate pH 8.0, 150 mM NaCl, and 5% (v/v) glycerol) and the column was washed using SEC buffer at a flow rate of 1 mL/min. The peak fractions were pooled, glycerol was added to a final concentration of 20%, and concentrated

using a 10 kDa Amicon centrifugal filter. Aliquots were frozen in liquid nitrogen, and stored at 80°C.

YlbL and YlbL-S234A were purified for *in vitro* assays as follows. 10xHis-Smt3-YlbL (a.a 36-341) and 10His-Smt3-YlbL-S234A (a.a. 36-341) were expressed from plasmids pPB157 and pPB181, respectively, as described above for antibody production. Cell pellets from a one liter culture were re-suspended in 30 mL lysis buffer (50 mM Tris pH 7.5, 250 mM NaCl, 10% sucrose, and 20 mM imidazole) and lysed via sonication. Cell lysates were clarified via centrifugation: 18,000 rpm (Sorvall SS-34 rotor) for 30 minutes at 4°C. Clarified lysates were applied to Ni^{2+}-NTA-agarose pre-equilibrated with lysis buffer. The column was washed with 20 column volumes wash buffer (25 mM Tris pH 8.0, 100 mM NaCl, 30 mM imidazole, and 5% glycerol). The Ni^{2+} column was eluted in four fractions of 1.2 column volumes using elution buffer (25 mM Tris pH 8.0, 100 mM NaCl, 10% glycerol, and 250 mM imidazole). Fractions 1-3 were pooled, DTT was added to 2.5 mM and 0.3 mg of Ulp1 was added. The elution was digested at 4°C overnight yielding untagged YlbL (a.a. 36-341) or untagged YlbL-S234A (a.a 36-341). The digest was desalted using a Zeba spin column into equilibration buffer (25 mM Tris pH 8.0, 50 mM NaCl, 5% glycerol, and 10 mM imidazole). The desalted digest was applied to a second column of Ni^{2+}-NTA-agarose and the flow-through was collected. The column was washed with one column volume of equilibration buffer. The flow-through and wash fractions were pooled and concentrated using a 10 kDa Amicon centrifugal filter. The concentrated protein was loaded onto a Sephacryl S-200 size exclusion column pre-equilibrated with SEC buffer (25 mM Tris pH 7.5, 5% glycerol, and 25 mM NaCl) and eluted over 1 column volume with SEC buffer at a flow rate of 1 ml/min. Peak fractions were pooled, glycerol was added to 20% and the

final protein was concentrated using a 10 kDa Amicon centrifugal filter. The concentrated protein was aliquoted, frozen in liquid nitrogen, and stored at -80°C.

YneA

10xHis-Smt3-YneA (a.a. 28-103) was expressed from pPB204 and harvested as described for YlbL above. Cell pellets were re-suspended in lysis buffer (50 mM Tris pH 7.5, 250 mM NaCl, 10% (w/v) sucrose, 20 mM imidazole). Cells were lysed via sonication and the lysate was clarified as described for YlbL above. The clarified lysate was incubated with Ni^{2+}-NTA-agarose beads, pre-equilibrated with lysis buffer, and incubated on rotator at 4°C for 1 hour. The lysate/bead slurry was loaded into a gravity flow column and the beads were allowed to settle for 5-10 minutes. The lysate was collected as the flow-through. The column was washed with 30 column volumes wash buffer (25 mM Tris pH 7.5, 150 mM NaCl, 5% (v/v) glycerol, and 20 mM imidazole). The column was eluted in 6 fractions of 1 column volume each of elution buffer (25 mM Tris pH 7.5, 150 mM NaCl, 10% (v/v) glycerol, 250 mM imidazole). The fractions with the highest protein concentration were pooled, DTT was added to 1 mM and digested with MBP-Ulp1 overnight at 4°C. The resulting digest, yielding untagged YneA (a.a. 28-103), was loaded onto a sephacryl S200 26/60 column pre-equilibrated with SEC buffer (25 mM Tris pH 8.0, 100 mM NaCl, and 5% (v/v) glycerol) and washed at a flow rate of 2 mL/min. The peak fractions were pooled, and glycerol was added to 20%. The protein was loaded onto a HiTrap Q column (GE life sciences), pre-equilibrated with 7.5% Q-finish buffer (25 mM Tris pH 8.0, 5% (v/v) glycerol, and 1 M NaCl) and 92.5% Q-start buffer (25 mM Tris pH 8.0, and 5% (v/v) glycerol). The column was washed with 5 column volumes 7.5% Q-finish buffer. The column was eluted with 30 column volumes over a linear gradient from 7.5% to 50% Q-finish buffer at a flow rate of 2 mL/min. Peak fractions were pooled, glycerol was added to a final concentration of 20%,

and the protein was concentrated using a 3 kDa Amicon centrifugal filter. Aliquots were frozen in liquid nitrogen and stored at -80°C.

CtpA and CtpA-S297A

Expression conditions for CtpA fusions were similar to previous reports for CtpB (15). MBP-Smt3-CtpA and MBP-Smt3-CtpA-S297A were expressed in BL21 pLysY/LacIq (NEBC3013) cells from pPB203 and pPB214, respectively. A starter culture of LB + 50 µg/mL kanamycin was inoculated using several colonies from a fresh transformation and incubated at 37°C with shaking (200 rpm) until an OD_{600} = 0.5. One liter LB + 50 µg/mL kanamycin cultures were inoculated by diluting the starter culture 1:50 and incubated at 37°C until an OD_{600} of about 0.7. Glucose was added to 0.2% and expression was induced by addition of IPTG to 1 mM and incubating at 37°C for 45 minutes. Cells were harvested via centrifugation: 4,000 *g* for 20 minutes at 4°C. Cell pellets were re-suspended in 30 mL lysis buffer (50 mM Tris pH 7.5, 250 mM NaCl, 10% (w/v) sucrose) per one liter of culture and lysed via sonication. Lysates were clarified via centrifugation: 12,500 rpm (Sorvall SS-34 rotor) for 45 minutes at 4°C. Clarified lysates were mixed with pre-equilibrated 2.5 mL amylose resin (NEB) on a rotator at 4°C. The lysate/resin mixture was loaded into a gravity flow column and the resin was allowed to settle for 5-10 minutes. The lysate was allowed to flow over the packed resin. The column was washed with 20 column volumes of wash buffer (25 mM Tris pH 8.0, 100 mM NaCl, 10%(v/v) glycerol). The column was eluted with 4 fractions of 3 mL each in elution buffer (25 mM Tris pH 8.0, 100 mM NaCl, 10%(v/v) glycerol, and 50 mM maltose). Fractions 1-3 were pooled, DTT was added to 2 mM, and digested with MBP-Ulp1 overnight at 4°C. The resulting digest, yielding untagged CtpA (or CtpA-S297A), was diluted two-fold with Q-start buffer (25 mM Tris, pH 8.0, 5% glycerol) to bring the NaCl concentration to 50 mM, and loaded onto a HiTrap Q-column pre-

equilibrated with 5% Q-finish buffer (25 mM Tris, pH 8.0, 1 M NaCl, 5% glycerol). The column was washed with 5 column volumes 5% Q-finish buffer. The column was eluted with 25 column volumes over a linear gradient from 5 to 50% Q finish buffer. The peak fractions were pooled, concentrated using a 30 kDa Amicon centrifugal filter, and loaded onto a sephacryl 16/60 S-200 column pre-equilibrated with SEC buffer (25 mM Tris pH 7.5, 25 mM NaCl, 5% glycerol). The column was washed with SEC buffer at a flow rate of 1 mL/min. Peak fractions were pooled, glycerol was added to 20%, and concentrated using a 30 kDa Amicon centrifugal filter. Aliquots were frozen in liquid nitrogen, and stored at -80°C.

6xHis-Ulp1

6xHis-Ulp1 was expressed from plasmid pPB13 in *E. coli* NiCo21 cells (NEBC2529H). One liter cultures of LB + 50 µg/mL kanamycin were inoculated with an overnight culture at 1:100 and grown at 37°C until OD_{600} of about 0.7. Cultures were induced with 0.5 mM IPTG and incubated at 37°C for 3 hours. Cells were pelleted via centrifugation: 4,000 *g* for 20 minutes at 4°C. Cells were re-suspended in lysis buffer (50 mM Tris pH 7.5, 300 mM NaCl, 5% (w/v) sucrose, and 20 mM imidazole) and lysed via sonication. Lysates were clarified via centrifugation: 18,000 rpm (Sorvall SS-34 rotor) for 45 minutes at 4°C. Clarified lysates were loaded onto Ni^{2+}-NTA-agarose beads pre-equilibrated with lysis buffer and incubated at 4°C for 1 hour on a rotator. The lysate was collected as the flow through and the column was washed with 25 column volumes wash buffer (25 mM Tris pH 7.5, 300 mM NaCl, and 20 mM imidazole). The column was eluted in elution buffer (25 mM Tris pH 7.5, 300 mM NaCl, and 250 mM imidazole). The most concentrated fractions were pooled; aliquots were frozen in liquid nitrogen, and stored at -80°C.

MBP-Ulp1

MBP-Ulp1 was expressed from pPB200 in *E. coli* BL21 DE3 cells. One liter LB + 50 µg/mL kanamycin cultures were inoculated with an overnight culture at 1:100. Cultures were grown at 37°C until OD_{600} of about 0.5, glucose was added to 0.2%, and then protein expression was induced by addition of 0.25 mM IPTG. Cultures were incubated at 20°C overnight. Cells were pelleted via centrifugation: 4,000 *g* for 20 minutes at 4°C. Cell pellets were re-suspended in lysis buffer (50 mM Tris pH 7.5, 250 mM NaCl, 10% (w/v) sucrose, and 1 mM DTT), and lysed via sonication. Lysates were cleared via centrifugation and MBP-Ulp1 was purified on an amylose column as described for CtpA above. Peak fractions from the amylose column were loaded onto a sephacryl S200 26/60 column, pre-equilibrated with SEC buffer (25 mM Tris pH 7.5, 200 mM NaCl, 2% glycerol). The column was washed with SEC buffer at a flow rate of 2 mL/min. Peak fractions were pooled, glycerol was added to 20%, and DTT was added to 1 mM. MBP-Ulp1 was concentrated to about 1.7 mg/mL, aliquots were frozen in liquid nitrogen and stored at -80°C.

Strain construction

General strain construction methods

Generation of competent *B. subtilis* cultures for generating new genotypes was performed as described below in “Transposon insertion mutant library construction,” or as previously reported (16).

Genome editing using CRISPR/Cas9 was performed as described previously (16, 17). *B. subtilis* strains were transformed with the indicated plasmid (prepared from *E. coli* MC1061), plated on LB agar + 100 µg/mL spectinomycin and incubated at 30°C overnight. Isolates were

colony purified by restreaking on LB agar + 100 µg/mL spectinomycin and incubating at 30°C overnight. The editing plasmid was evicted by restreaking isolates on LB agar and incubating at 45°C for 8-12 hours or overnight. Loss of the editing plasmid was verified by restreaking LB agar + 100 µg/mL spectinomycin and on LB agar. Isolates that were unable to grow in the presence of spectinomycin were used for PCR genotyping.

Gene deletions using the *B. subtilis* knockout library were performed as described (18). Chromosomal DNA, extracted as described below (see extraction of PY79 chromosomal DNA), was used to transform PY79 or the indicated strain. Incorporation of the *erm* cassette at the appropriate locus was verified via PCR genotyping. Removal of the *erm* cassette was performed following transformation with pDR244, which contains *cre* recombinase. Eviction of pDR244 was performed as described for a CRISPR/Cas9 genome editing plasmid. Loss of the *erm* cassette was verified by sensitivity to erythromycin and PCR genotyping.

Integration of inducible constructs at the *amyE* locus was achieved via double crossover recombination. For constructs containing a xylose inducible promoter (P_{xyl}), strains were transformed with plasmids that had been digested with two unique restriction enzymes (KpnI-HF and ScaI-HF) or with genomic DNA of a strain already generated (see detailed strain construction) and transformants were selected using LB agar + 5 µg/mL chloramphenicol. Isolates were colony purified by restreaking on LB agar + 5 µg/mL chloramphenicol. Incorporation via double cross-over at *amyE* was determined by screening for an inability to utilize starch and by testing for the absence of a spectinomycin resistance cassette that is present on part of the plasmid that is not integrated. For constructs containing an IPTG inducible promoter (P_{hyp}), strains were transformed with plasmids that had been digested with two unique restriction enzymes (SpeI and SacI-HF) and transformants were selected using LB agar + 100

μg/mL spectinomycin. Isolates were colony purified by restreaking on LB agar + 100 μg/mL spectinomycin. Incorporation via double cross-over at *amyE* was determined by screening for an inability to utilize starch.

Individual strain construction

PEB85 (Δ*recN*::*erm*): PY79 was transformed with genomic DNA isolated from PEB75. Replacement of *recN* with the *erm* cassette was verified via PCR genotyping using oPEB54/55.

PEB235 (Δ*recR*): PY79 was edited using CRISPR/Cas9 genome editing with plasmid pPB46. Deletion of recR was verified via PCR genotyping using oPEB243/246.

PEB278 (Δ*rnhC*): PEB278 is a clonal isolate of JWS224, which was generated by deletion of *rnhC* from PY79 using the pMiniMAD method as described previously (16).

PEB307 (Δ*uvrA*): PY79 was edited using CRISPR/Cas9 genome editing with plasmid pPB82. Deletion of *uvrA* was verified via PCR genotyping using oPEB423/424.

PEB308 (Δ*uvrB*): PY79 was edited using CRISPR/Cas9 genome editing with plasmid pPB83. Deletion of *uvrB* was verified via PCR genotyping using oPEB432/433.

PEB310 (Δ*uvrC*): PY79 was edited using CRISPR/Cas9 genome editing with plasmid pPB85. Deletion of *uvrC* was verified via PCR genotyping using oPEB443/444.

PEB316 (Δ*yprA*): PY79 was edited using CRISPR/Cas9 genome editing with plasmid pPB86. Deletion of *yprA* was verified via PCR genotyping using oPEB452/453.

PEB318 (Δ*yprB*): PY79 was edited using CRISPR/Cas9 genome editing with plasmid pPB87. Deletion of *yprB* was verified via PCR genotyping using oPEB461/462.

PEB322 (Δ*ylbK*): PY79 was edited using CRISPR/Cas9 genome editing with plasmid pPB98. Deletion of *ylbK* was verified via PCR genotyping using oPEB472/473.

PEB324 (Δ*ylbL*): PY79 was edited using CRISPR/Cas9 genome editing with plasmid pPB99. Deletion of *ylbL* was verified via PCR genotyping using oPEB481/482.

PEB334 (Δ*sodA*): PY79 was edited using CRISPR/Cas9 genome editing with plasmid pPB103. Deletion of *sodA* was verified via PCR genotyping using oPEB510/511.

PEB346: PY79 was transformed with plasmid pPB111. This strain was only used to generate other strains using extracted genomic DNA.

PEB349: PEB324 was transformed with plasmid pPB108. This strain was only used to generate other strains using extracted genomic DNA.

PEB350: PY79 was transformed with plasmid pPB107. This strain was only used to generate other strains using extracted genomic DNA.

PEB353 (Δ*queA*): PY79 was edited using CRISPR/Cas9 genome editing with plasmid pPB120. Deletion of *queA* was verified via PCR genotyping using oPEB583/584.

PEB355 (Δ*ctpA*): PY79 was edited using CRISPR/Cas9 genome editing with plasmid pPB121. Deletion of *ctpA* was verified via PCR genotyping using oPEB592/593.

PEB357 (Δ*ysoA*): PY79 was edited using CRISPR/Cas9 genome editing with plasmid pPB122. Deletion of *ysoA* was verified via PCR genotyping using oPEB601/602.

PEB373 (Δ*ylbK, amyE::Pxyl-ylbK*): PEB322 was transformed with genomic DNA from PEB350. Retention of the Δ*ylbK* allele was verified via PCR genotyping oPEB472/473.

PEB375 (Δ*ylbL, amyE::Pxyl-ylbL*): PEB324 was transformed with genomic DNA from PEB349.

PEB377 (Δ*ylbL, amyE::Pxyl-ylbL-S243A*): PEB324 was transformed with genomic DNA from PEB346. Retention of the Δ*ylbL* allele was verified via PCR genotyping using oPEB481/482.

PEB382 (Δ*ylmE*): PY79 was edited using CRISPR/Cas9 genome editing with plasmid pPB123 as a Gibson assembly reaction because the plasmid was not able to be isolated from *E. coli*. Deletion of *ylmE* was verified via PCR genotyping using oPEB610/611.

PEB384 (Δ*sepF*): PY79 was edited using CRISPR/Cas9 genome editing with plasmid pPB124 as a Gibson assembly reaction because the plasmid was not able to be isolated from *E. coli*. Deletion of *sepF* was verified via PCR genotyping using oPEB612/623.

PEB386 (Δ*ylmG*): PY79 was edited using CRISPR/Cas9 genome editing with plasmid pPB125 as a Gibson assembly reaction because the plasmid was not able to be isolated from *E. coli*. Deletion of *ylmG* was verified via PCR genotyping using oPEB612/623.

PEB390 (Δ*ylbK, amyE::*P_{xyl}*-ylbL*): PEB322 was transformed with genomic DNA from PEB349. Retention of the Δ*ylbK* allele was verified via PCR genotyping using oPEB472/473. A wild-type *ylbL* locus was verified via PCR genotyping using oPEB481/482.

PEB392 (Δ*ylbK, amyE::*P_{xyl}*-ylbL-S243A*): PEB322 was transformed with genomic DNA from PEB346. Retention of the Δ*ylbK* allele was verified via PCR genotyping using oPEB472/473.

PEB418 (Δ*ytmP*): PY79 was edited using CRISPR/Cas9 genome editing with plasmid pPB140. Deletion of *ytmP* was verified via PCR genotyping using oPEB644/645.

PEB420 (Δ*bcrC*): PY79 was edited using CRISPR/Cas9 genome editing with plasmid pPB141. Deletion of *bcrC* was verified via PCR genotyping using oPEB653/654.

PEB422 (Δ*ecsA*): PY79 was edited using CRISPR/Cas9 genome editing with plasmid pPB142. Deletion of *ecsA* was verified via PCR genotyping using oPEB662/663.

PEB424 (Δ*ecsB*): PY79 was edited using CRISPR/Cas9 genome editing with plasmid pPB143. Deletion of *ecsB* was verified via PCR genotyping using oPEB671/672.

PEB427 (Δ*ylbK-2*): PY79 was edited using CRISPR/Cas9 genome editing with plasmid pPB149. Deletion of *ylbK-2* (deletion of amino acids 4-246; leaving amino acids 1-3 and 247-260) was verified via PCR genotyping using oPEB472/473.

PEB433 (Δ*yneA::erm*): PY79 was transformed using genomic DNA from PEB 432. Replacement of *yneA* with the *erm* cassette was verified via PCR genotyping using oPEB492/493.

PEB436 (Δ*ylbL,* Δ*yneA::erm*): PEB433 was edited using CRISPR/Cas9 genome editing with plasmid pPB99. Deletion of *ylbL* was verified via PCR genotyping using oPEB481/482.

PEB439 (Δ*yneA::loxP*): PEB433 was transformed with pDR244, which was then evicted.

PEB441 (Δ*ylbL,* Δ*yneA::loxP*): PEB436 was transformed with pDR244, which was then evicted.

PEB461 (Δ*crh::erm*): PY79 was transformed using genomic DNA from PEB 449. Replacement of *crh* with the *erm* cassette was verified via PCR genotyping using oPEB780/781.

PEB463 (Δ*lgt::erm*): PY79 was transformed using genomic DNA from PEB 450. Replacement of *lgt* with the *erm* cassette was verified via PCR genotyping using oPEB780/781.

PEB465 (Δ*crh::erm*): PY79 was transformed using genomic DNA from PEB 449. Replacement of *crh* with the *erm* cassette was verified via PCR genotyping using oPEB782/783.

PEB467 (Δ*sdaAB::erm*): PY79 was transformed using genomic DNA from PEB 452. Replacement of *sdaAB* with the *erm* cassette was verified via PCR genotyping using oPEB788/789.

PEB469 (Δ*ydzU::erm*): PY79 was transformed using genomic DNA from PEB 453. Replacement of *ydzU* with the *erm* cassette was verified via PCR genotyping using oPEB790/791.

PEB471 (Δ*radA::erm*): PY79 was transformed using genomic DNA from PEB 454. Replacement of *radA* with the *erm* cassette was verified via PCR genotyping using oPEB792/793.

PEB473 (Δ*cymR::erm*): PY79 was transformed using genomic DNA from PEB 455. Replacement of *cymR* with the *erm* cassette was verified via PCR genotyping using oPEB794/795.

PEB475 (Δ*ywrC::erm*): PY79 was transformed using genomic DNA from PEB 456. Replacement of *ywrC* with the *erm* cassette was verified via PCR genotyping using oPEB796/797.

PEB478 (Δ*recG::erm*): PY79 was transformed using genomic DNA from PEB 477. Replacement of *recG* with the *erm* cassette was verified via PCR genotyping using oPEB786/787.

PEB482 (Δ*walH*): PY79 was edited using CRISPR/Cas9 genome editing with plasmid pPB174. Deletion of *walH* was verified via PCR genotyping using oPEB744/745.

PEB485 (Δ*yycI*): PY79 was edited using CRISPR/Cas9 genome editing with plasmid pPB175. Deletion of *yycI* was verified via PCR genotyping using oPEB753/754.

PEB488 (Δ*walJ*): PY79 was edited using CRISPR/Cas9 genome editing with plasmid pPB176. Deletion of *walJ* was verified via PCR genotyping using oPEB762/763.

PEB491 (Δ*ruvB*): PY79 was edited using CRISPR/Cas9 genome editing with plasmid pPB179. Deletion of *ruvB* was verified via PCR genotyping using oPEB804/805.

PEB493 (Δ*ripX*): PY79 was edited using CRISPR/Cas9 genome editing with plasmid pPB180. Deletion of *ripX* was verified via PCR genotyping using oPEB813/814.

PEB533 (Δ*ylbL, amyE::*P_{xyl}*-ylbL-K279A*): PEB324 was transformed with plasmid pPB173.

PEB551 (Δ*ponA*): PY79 was transformed using genomic DNA from PEB 535. Replacement of *ponA* with the *erm* cassette was verified via PCR genotyping using oPEB574/575.

PEB555 (Δ*ylbL,* Δ*ctpA*): PEB324 was edited using CRISPR/Cas9 genome editing with plasmid pPB121. Deletion of *ctpA* was verified via PCR genotyping using oPEB592/593.

PEB557 (Δ*ylbL,* Δ*ctpA, amyE::*P_{xyl}*-ylbL*): PEB375 was edited using CRISPR/Cas9 genome editing with plasmid pPB121. Deletion of *ctpA* was verified via PCR genotyping using oPEB592/593.

PEB559 (Δ*yneA::loxP,* Δ*ctpA*): PEB439 was edited using CRISPR/Cas9 genome editing with plasmid pPB121. Deletion of *ctpA* was verified via PCR genotyping using oPEB592/593.

PEB561 (Δ*ylbL,* Δ*ctpA*, Δ*yneA::loxP*): PEB441 was edited using CRISPR/Cas9 genome editing with plasmid pPB121. Deletion of *ctpA* was verified via PCR genotyping using oPEB592/593.

PEB585 (Δ*uvrA*, Δ*yneA::loxP*): PEB439 was edited using CRISPR/Cas9 genome editing with plasmid pPB82. Deletion of *uvrA* was verified via PCR genotyping using oPEB423/424.

PEB595 (Δ*ylbL*, *amyE::*P_{xyl}*-ctpA*): PEB324 was transformed with plasmid pPB184.

PEB597 (Δ*ylbL*, *amyE::*P_{xyl}*-ctpA-S297A*): PEB324 was transformed with plasmid pPB185.

PEB599 (Δ*ctpA*, *amyE::*P_{xyl}*-ctpA*): PEB355 was transformed with plasmid pPB184.

PEB601 (Δ*ctpA*, *amyE::*P_{xyl}*-ctpA-S297A*): PEB355 was transformed with plasmid pPB185.

PEB603 (Δ*ctpA*, *amyE::*P_{xyl}*-ctpA-K322A*): PEB355 was transformed with plasmid pPB186.

PEB605 (Δ*ctpA*, *amyE::*P_{xyl}*-ylbL*): PEB355 was transformed with plasmid pPB108.

PEB607 (Δ*ctpA*, *amyE::*P_{xyl}*-ylbL-S234A*): PEB355 was transformed with plasmid pPB111.

PEB619 (Δ*ylbL*, Δ*ctpA*, *amyE::*P_{xyl}*-ctpA*): PEB555 was transformed with plasmid pPB184.

PEB621 (Δ*ylbL*, Δ*ctpA*, *amyE::*P_{xyl}*-ctpA-S297A*): PEB555 was transformed with plasmid pPB185.

PEB623 (Δ*ylbL*, Δ*ctpA*, *amyE::*P_{xyl}*-ylbL-S234A*): PEB555 was transformed with plasmid pPB111.

PEB677 (*amyE::*P_{hyp}*-yneA*): PY79 was transformed with plasmid pPB194.

PEB681 (Δ*ylbL*, *amyE::*P_{hyp}*-yneA*): PEB324 was transformed with plasmid pPB194.

PEB685 (Δ*ctpA*, *amyE::*P_{hyp}*-yneA*): PEB355 was transformed with plasmid pPB194.

PEB689 (Δ*ylbL*, Δ*ctpA*, *amyE::*P_{hyp}*-yneA*): PEB555 was transformed with plasmid pPB194.

PEB778 (Δ*fhuG*): PY79 was edited using CRISPR/Cas9 genome editing with plasmid pPB237. Deletion of *fhuG* was verified via PCR genotyping using oPEB918/919.

PEB780 (Δ*ylbL,* Δ*ctpA,* Δ*fhuG*): PEB555 was edited using CRISPR/Cas9 genome editing with plasmid pPB237. Deletion of *fhuG* was verified via PCR genotyping using oPEB918/919.

PEB782 (Δ*yfkH*): PY79 was edited using CRISPR/Cas9 genome editing with plasmid pPB238. Deletion of *yfkH* was verified via PCR genotyping using oPEB927/928.

PEB784 (Δ*ylbL,* Δ*ctpA,* Δ*yfkH*): PEB555 was edited using CRISPR/Cas9 genome editing with plasmid pPB238. Deletion of *yfkH* was verified via PCR genotyping using oPEB927/928.

PEB786 (Δ*ltaSA*): PY79 was edited using CRISPR/Cas9 genome editing with plasmid pPB239. Deletion of *ltaSA* was verified via PCR genotyping using oPEB936/937.

PEB788 (Δ*ylbL,* Δ*ctpA,* Δ*ltaSA*): PEB555 was edited using CRISPR/Cas9 genome editing with plasmid pPB239. Deletion of *ltaSA* was verified via PCR genotyping using oPEB936/937.

PEB790 (Δ*ykgA*): PY79 was edited using CRISPR/Cas9 genome editing with plasmid pPB240. Deletion of *ykgA* was verified via PCR genotyping using oPEB945/946.

PEB792 (Δ*ylbL,* Δ*ctpA,* Δ*ykgA*): PEB555 was edited using CRISPR/Cas9 genome editing with plasmid pPB240. Deletion of *ykgA* was verified via PCR genotyping using oPEB945/946.

PEB794 (Δ*cgeE*): PY79 was edited using CRISPR/Cas9 genome editing with plasmid pPB241. Deletion of *cgeE* was verified via PCR genotyping using oPEB954/955.

PEB796 (Δ*ylbL,* Δ*ctpA,* Δ*cgeE*): PEB555 was edited using CRISPR/Cas9 genome editing with plasmid pPB241. Deletion of *cgeE* was verified via PCR genotyping using oPEB954/955.

PEB798 (Δ*ysnF*): PY79 was edited using CRISPR/Cas9 genome editing with plasmid pPB242. Deletion of *ysnF* was verified via PCR genotyping using oPEB963/964.

PEB800 (Δ*ylbL,* Δ*ctpA,* Δ*ysnF*): PEB555 was edited using CRISPR/Cas9 genome editing with plasmid pPB242. Deletion of *ysnF* was verified via PCR genotyping using oPEB963/964.

PEB802 (Δ*lytG*): PY79 was edited using CRISPR/Cas9 genome editing with plasmid pPB243. Deletion of *lytG* was verified via PCR genotyping using oPEB972/973.

PEB804 (Δ*ylbL,* Δ*ctpA,* Δ*lytG*): PEB555 was edited using CRISPR/Cas9 genome editing with plasmid pPB243. Deletion of *lytG* was verified via PCR genotyping using oPEB972/973.

PEB806 (Δ*yxaB*): PY79 was edited using CRISPR/Cas9 genome editing with plasmid pPB244. Deletion of *yxaB* was verified via PCR genotyping using oPEB981/982.

PEB808 (Δ*ylbL,* Δ*ctpA,* Δ*yxaB*): PEB555 was edited using CRISPR/Cas9 genome editing with plasmid pPB244. Deletion of *yxaB* was verified via PCR genotyping using oPEB981/982.

PEB810 (Δ*ylbL,* Δ*ctpA,* Δ*ytmP*): PEB555 was edited using CRISPR/Cas9 genome editing with plasmid pPB140. Deletion of *ytmP* was verified via PCR genotyping using oPEB644/645.

Plasmid construction

General cloning techniques

Plasmids were assembled using Gibson assembly (19). Gibson assembly reactions were 10 or 12 μL consisting of 1x Gibson assembly master mix (0.1 M Tris pH 8.0, 5% PEG-8000, 10 mM $MgCl_2$, 10 mM DTT, 0.2 mM dNTPs, 1 mM NAD^+, 4 units/mL T_5 exonuclease, 25 units/mL Phusion DNA polymerase, 4,000 units/mL Taq DNA ligase) and 40-100 ng of each PCR product

and incubated at 50°C for 60 minutes or 90 minutes for CRISPR/Cas9 editing plasmids. All PCR products were isolated via gel extraction from an agarose gel. Gibson assembly reactions were used to transform Top10 or MC1061 *E. coli*.

Plasmids that were intermediate products of editing plasmids containing only the targeting spacer were generated by ligation of the proto-spacer into pPB41 as described previously (16, 17). Briefly, pPB41 was digested with BsaI-HF (NEB), and then treated with CIP (NEB). The digestion product was purified by gel extraction from an agarose gel. Proto-spacers were annealed in 1x annealing buffer (10 mM Tris, pH 8.0, 100 mM NaCl, 10 µM EDTA) at a concentration of 10 µM each by incubation in a 100°C heat block for 5 minutes, followed by transferring to a beaker of water pre-heated to 100°C. The annealing reactions were allowed to cool slowly to room temperature in the beaker of water. The annealed proto-spacers were phosphorylated using T_4 PNK (NEB). The phosphorylated proto-spacers were ligated to digested pPB41 using T_4 DNA ligase (NEB). The resulting ligations were used to transform Top10 or MC1061 *E. coli*. Plasmids sequences were verified by Sanger sequencing using oPEB253.

Individual plasmid construction

pPB44: A proto-spacer targeting *recR* (oPEB241/242) was ligated to pPB41.

pPB46: The upstream and downstream portions of the Δ*recR* editing template were PCR amplified using oPEB286/244 and oPEB245/287, respectively. CRISPR/Cas9 was PCR amplified using oPEB232/234 and pPB46 as the template. The pPB41 vector was PCR amplified using oPEB217/218. These four PCR products were used in a Gibson assembly reaction to generate a Δ*recR* editing plasmid. Clones were verified by Sanger sequencing with oPEB227 and oPEB253.

pPB47: pBR322 was PCR amplified using oPEB267/268, and the spectinomycin resistance cassette was PCR amplified from pDR110 using oPEB269/270. The resulting PCR products were used in a Gibson assembly reaction generating a plasmid in which the tetracycline resistance cassette of pBR322 had been replaced with the spectinomycin resistance cassette of pDR110.

pPB72: A proto-spacer targeting *uvrA* (oPEB417/418) was ligated to pPB41.

pPB73: A proto-spacer targeting *uvrB* (oPEB426/427) was ligated to pPB41.

pPB74: A proto-spacer targeting *uvrC* (oPEB437/438) was ligated to pPB41.

pPB75: A proto-spacer targeting *yprA* (oPEB446/447) was ligated to pPB41.

pPB76: A proto-spacer targeting *yprB* (oPEB455/456) was ligated to pPB41.

pPB77: A proto-spacer targeting *ylbK* (oPEB466/467) was ligated to pPB41.

pPB78: A proto-spacer targeting *ylbL* (oPEB475/476) was ligated to pPB41.

pPB81: A proto-spacer targeting *sodA* (oPEB504/505) was ligated to pPB41.

pPB82: The upstream and downstream portions of the Δ*uvrA* editing template were PCR amplified using oPEB419/420 and oPEB421/422, respectively. CRISPR/Cas9 was PCR amplified using oPEB232/234 and pPB72 as the template. The pPB41 vector was PCR amplified using oPEB217/218. These four PCR products were used in a Gibson assembly reaction to generate a Δ*uvrA* editing plasmid. Clones were verified by Sanger sequencing with oPEB227, oPEB253, and oPEB425.

pPB83: The upstream and downstream portions of the Δ*uvrB* editing template were PCR amplified using oPEB428/429 and oPEB430/431, respectively. CRISPR/Cas9 was PCR amplified using oPEB232/234 and pPB73 as the template. The pPB41 vector was PCR amplified using oPEB217/218. These four PCR products were used in a Gibson assembly reaction to generate a Δ*uvrB* editing plasmid. Clones were verified by Sanger sequencing with oPEB227, oPEB253, and oPEB434.

pPB85: The upstream and downstream portions of the Δ*uvrC* editing template were PCR amplified using oPEB439/440 and oPEB441/442, respectively. CRISPR/Cas9 was PCR amplified using oPEB232/234 and pPB74 as the template. The pPB41 vector was PCR amplified using oPEB217/218. These four PCR products were used in a Gibson assembly reaction to generate a Δ*uvrC* editing plasmid. Clones were verified by Sanger sequencing with oPEB227, oPEB253, and oPEB445.

pPB86: The upstream and downstream portions of the Δ*yprA* editing template were PCR amplified using oPEB448/449 and oPEB450/451, respectively. CRISPR/Cas9 was PCR amplified using oPEB232/234 and pPB75 as the template. The pPB41 vector was PCR amplified using oPEB217/218. These four PCR products were used in a Gibson assembly reaction to generate a Δ*yprA* editing plasmid. Clones were verified by Sanger sequencing with oPEB227, oPEB253, and oPEB454.

pPB87: The upstream and downstream portions of the Δ*yprB* editing template were PCR amplified using oPEB457/458 and oPEB459/460, respectively. CRISPR/Cas9 was PCR amplified using oPEB232/234 and pPB76 as the template. The pPB41 vector was PCR amplified using oPEB217/218. These four PCR products were used in a Gibson assembly reaction to

generate a Δ*yprB* editing plasmid. Clones were verified by Sanger sequencing with oPEB227, oPEB253, and oPEB463.

pPB98: The upstream and downstream portions of the Δ*ylbK* editing template were PCR amplified using oPEB468/469 and oPEB470/471, respectively. CRISPR/Cas9 was PCR amplified using oPEB232/234 and pPB77 as the template. The pPB41 vector was PCR amplified using oPEB217/218. These four PCR products were used in a Gibson assembly reaction to generate a Δ*ylbK* editing plasmid. Clones were verified by Sanger sequencing with oPEB227, oPEB253, and oPEB474.

pPB99: The upstream and downstream portions of the Δ*ylbL* editing template were PCR amplified using oPEB477/478 and oPEB479/480, respectively. CRISPR/Cas9 was PCR amplified using oPEB232/234 and pPB78 as the template. The pPB41 vector was PCR amplified using oPEB217/218. These four PCR products were used in a Gibson assembly reaction to generate a Δ*ylbL* editing plasmid. Clones were verified by Sanger sequencing with oPEB227, oPEB253, and oPEB483.

pPB103: The upstream and downstream portions of the Δ*sodA* editing template were PCR amplified using oPEB506/507 and oPEB508/509, respectively. CRISPR/Cas9 was PCR amplified using oPEB232/234 and pPB81 as the template. The pPB41 vector was PCR amplified using oPEB217/218. These four PCR products were used in a Gibson assembly reaction to generate a Δ*sodA* editing plasmid. Clones were verified by Sanger sequencing with oPEB227, oPEB253, and oPEB512.

pPB107: The upstream portion of *amyE* and the P_{xyl} promoter were PCR amplified using oPEB370/383. The chloramphenicol resistance cassette and the downstream portion of *amyE*

were amplified using oPEB557/377. pPB47 was PCR amplified using oPEB116/117. The open reading frame (ORF) of *ylbK* was PCR amplified using oPEB558/559. These four PCR products were used in a Gibson assembly reaction to generate a plasmid to integrate *ylbK* under the control of P_{xyl} at the *amyE* locus. Clones were verified by Sanger sequencing with oPEB345 and oPEB348.

pPB108: The upstream portion of *amyE* and the P_{xyl} promoter were PCR amplified using oPEB370/383. The chloramphenicol resistance cassette and the downstream portion of *amyE* were amplified using oPEB557/377. pPB47 was PCR amplified using oPEB116/117. The ORF of *ylbL* was PCR amplified using oPEB560/561. These four PCR products were used in a Gibson assembly reaction to generate a plasmid to integrate *ylbL* under the control of P_{xyl} at the *amyE* locus. Clones were verified by Sanger sequencing with oPEB345 and oPEB348.

pPB111: The upstream portion of *amyE* and the P_{xyl} promoter were PCR amplified using oPEB370/383. The chloramphenicol resistance cassette and the downstream portion of *amyE* were amplified using oPEB557/377. Plasmid pPB47 was PCR amplified using oPEB116/117. The upstream portion of the *ylbL* ORF containing the *S234A* mutation was PCR amplified using oPEB560/567. The downstream portion of the *ylbL* ORF containing the *S234A* mutation was PCR amplified using oPEB566/561. These five PCR products were used in a Gibson assembly reaction to generate a plasmid to integrate *ylbL-S234A* under the control of P_{xyl} at the *amyE* locus. Clones were verified by Sanger sequencing with oPEB345 and oPEB348.

pPB113: A proto-spacer targeting *queA* (oPEB577/578) was ligated to pPB41.

pPB114: A proto-spacer targeting *ctpA* (oPEB586/587) was ligated to pPB41.

pPB115: A proto-spacer targeting *ysoA* (oPEB595/596) was ligated to pPB41.

pPB116: A proto-spacer targeting *ylmE* (oPEB604/605) was ligated to pPB41.

pPB117: A proto-spacer targeting *sepF* (oPEB613/614) was ligated to pPB41.

pPB118: A proto-spacer targeting *ylmG* (oPEB618/619) was ligated to pPB41.

pPB120: The upstream and downstream portions of the Δ*queA* editing template were PCR amplified using oPEB579/580 and oPEB581/582, respectively. CRISPR/Cas9 was PCR amplified using oPEB232/234 and pPB113 as the template. The pPB41 vector was PCR amplified using oPEB217/218. These four PCR products were used in a Gibson assembly reaction to generate a Δ*queA* editing plasmid. Clones were verified by Sanger sequencing with oPEB227, oPEB253, and oPEB585.

pPB121: The upstream and downstream portions of the Δ*ctpA* editing template were PCR amplified using oPEB588/589 and oPEB590/591, respectively. CRISPR/Cas9 was PCR amplified using oPEB232/234 and pPB114 as the template. The pPB41 vector was PCR amplified using oPEB217/218. These four PCR products were used in a Gibson assembly reaction to generate a Δ*ctpA* editing plasmid. Clones were verified by Sanger sequencing with oPEB227, oPEB253, and oPEB594.

pPB122: The upstream and downstream portions of the Δ*ysoA* editing template were PCR amplified using oPEB597/598 and oPEB599/600, respectively. CRISPR/Cas9 was PCR amplified using oPEB232/234 and pPB115 as the template. The pPB41 vector was PCR amplified using oPEB217/218. These four PCR products were used in a Gibson assembly reaction to generate a Δ*ysoA* editing plasmid. Clones were verified by Sanger sequencing with oPEB227, oPEB253, and oPEB603.

pPB123: The upstream and downstream portions of the Δ*ylmE* editing template were PCR amplified using oPEB606/607 and oPEB608/609, respectively. CRISPR/Cas9 was PCR amplified using oPEB232/234 and pPB116 as the template. The pPB41 vector was PCR amplified using oPEB217/218. These four PCR products were used in a Gibson assembly reaction to generate a Δ*ylmE* editing plasmid. The Gibson assembly reaction was used to transform PY79.

pPB124: The upstream and downstream portions of the Δ*sepF* editing template were PCR amplified using oPEB606/615 and oPEB616/609, respectively. CRISPR/Cas9 was PCR amplified using oPEB232/234 and pPB117 as the template. The pPB41 vector was PCR amplified using oPEB217/218. These four PCR products were used in a Gibson assembly reaction to generate a Δ*sepF* editing plasmid. The Gibson assembly reaction was used to transform PY79.

pPB125: The upstream and downstream portions of the Δ*ylmG* editing template were PCR amplified using oPEB606/620 and oPEB621/609, respectively. CRISPR/Cas9 was PCR amplified using oPEB232/234 and pPB118 as the template. The pPB41 vector was PCR amplified using oPEB217/218. These four PCR products were used in a Gibson assembly reaction to generate a Δ*ylmG* editing plasmid. The Gibson assembly reaction was used to transform PY79.

pPB130: A proto-spacer targeting *ytmP* (oPEB638/639) was ligated to pPB41.

pPB131: A proto-spacer targeting *bcrC* (oPEB647/648) was ligated to pPB41.

pPB132: A proto-spacer targeting *ecsA* (oPEB656/657) was ligated to pPB41.

pPB133: A proto-spacer targeting *ecsB* (oPEB665/666) was ligated to pPB41.

pPB140: The upstream and downstream portions of the Δ*ytmP* editing template were PCR amplified using oPEB640/641 and oPEB642/643, respectively. CRISPR/Cas9 was PCR amplified using oPEB232/234 and pPB130 as the template. The pPB41 vector was PCR amplified using oPEB217/218. These four PCR products were used in a Gibson assembly reaction to generate a Δ*ytmP* editing plasmid. Clones were verified by Sanger sequencing with oPEB227, oPEB253, and oPEB646.

pPB141: The upstream and downstream portions of the Δ*bcrC* editing template were PCR amplified using oPEB649/650 and oPEB651/652, respectively. CRISPR/Cas9 was PCR amplified using oPEB232/234 and pPB131 as the template. The pPB41 vector was PCR amplified using oPEB217/218. These four PCR products were used in a Gibson assembly reaction to generate a Δ*bcrC* editing plasmid. Clones were verified by Sanger sequencing with oPEB227, oPEB253, and oPEB655.

pPB142: The upstream and downstream portions of the Δ*ecsA* editing template were PCR amplified using oPEB658/659 and oPEB660/661, respectively. CRISPR/Cas9 was PCR amplified using oPEB232/234 and pPB132 as the template. The pPB41 vector was PCR amplified using oPEB217/218. These four PCR products were used in a Gibson assembly reaction to generate a Δ*ecsA* editing plasmid. Clones were verified by Sanger sequencing with oPEB227, oPEB253, and oPEB664.

pPB143: The upstream and downstream portions of the Δ*ecsB* editing template were PCR amplified using oPEB667/668 and oPEB669/670, respectively. CRISPR/Cas9 was PCR amplified using oPEB232/234 and pPB133 as the template. The pPB41 vector was PCR

amplified using oPEB217/218. These four PCR products were used in a Gibson assembly reaction to generate a Δ*ecsB* editing plasmid. Clones were verified by Sanger sequencing with oPEB227, oPEB253, and oPEB673.

pPB149: The upstream and downstream portions of the Δ*ylbK-2* (deletion of all but the codons for the first 3 and the last 14 amino acids) editing template were PCR amplified using oPEB468/709 and oPEB710/471, respectively. CRISPR/Cas9 was PCR amplified using oPEB232/234 and pPB77 as the template. The pPB41 vector was PCR amplified using oPEB217/218. These four PCR products were used in a Gibson assembly reaction to generate a Δ*ylbK-2* editing plasmid. Clones were verified by Sanger sequencing with oPEB227, oPEB253, and oPEB474.

pPB154: A proto-spacer targeting *walH* (oPEB738/739) was ligated to pPB41.

pPB155: A proto-spacer targeting *yycI* (oPEB747/748) was ligated to pPB41.

pPB156: A proto-spacer targeting *walJ* (oPEB756/757) was ligated to pPB41.

pPB157: The ORF coding for a.a. 36-341 of YlbL was PCR amplified using oPEB773/677. The plasmid pPB12 was PCR amplified using oPEB56/57. These two PCR products were used in a Gibson assembly to generate a plasmid for overexpression of a 10xHis-Smt3-YlbL(36-341) fusion protein in *E. coli*. Clones were verified via Sanger sequencing using oPEB527 and oPEB58.

pPB173: The upstream portion of *amyE* and the P_{xyl} promoter were PCR amplified using oPEB370/383. The chloramphenicol resistance cassette and the downstream portion of *amyE* were amplified using oPEB557/377. pPB47 was PCR amplified using oPEB116/117. The

upstream portion of the *ylbL* ORF containing the *K279A* mutation was PCR amplified using oPEB560/772. The downstream portion of the *ylbL* ORF containing the *K279A* mutation was PCR amplified using oPEB771/561. These five PCR products were used in a Gibson assembly reaction to generate a plasmid to integrate *ylbL-K279A* under the control of P_{xyl} at the *amyE* locus. Clones were verified by Sanger sequencing with oPEB345 and oPEB348.

pPB174: The upstream and downstream portions of the Δ*walH* editing template were PCR amplified using oPEB740/741 and oPEB742/743, respectively. CRISPR/Cas9 was PCR amplified using oPEB232/234 and pPB154 as the template. The pPB41 vector was PCR amplified using oPEB217/218. These four PCR products were used in a Gibson assembly reaction to generate a Δ*walH* editing plasmid. Clones were verified by Sanger sequencing with oPEB227, oPEB253, and oPEB746.

pPB175: The upstream and downstream portions of the Δ*yycI* editing template were PCR amplified using oPEB749/750 and oPEB751/752, respectively. CRISPR/Cas9 was PCR amplified using oPEB232/234 and pPB155 as the template. The pPB41 vector was PCR amplified using oPEB217/218. These four PCR products were used in a Gibson assembly reaction to generate a Δ*yycI* editing plasmid. Clones were verified by Sanger sequencing with oPEB227, oPEB253, and oPEB755.

pPB176: The upstream and downstream portions of the Δ*walJ* editing template were PCR amplified using oPEB758/759 and oPEB760/761, respectively. CRISPR/Cas9 was PCR amplified using oPEB232/234 and pPB156 as the template. The pPB41 vector was PCR amplified using oPEB217/218. These four PCR products were used in a Gibson assembly

reaction to generate a Δ*walJ* editing plasmid. Clones were verified by Sanger sequencing with oPEB227, oPEB253, and oPEB764.

pPB177: A proto-spacer targeting *ruvB* (oPEB798/799) was ligated to pPB41.

pPB178: A proto-spacer targeting *ripX* (oPEB807/808) was ligated to pPB41.

pPB179: The upstream and downstream portions of the Δ*ruvB* editing template were PCR amplified using oPEB800/801 and oPEB802/803, respectively. CRISPR/Cas9 was PCR amplified using oPEB232/234 and pPB177 as the template. The pPB41 vector was PCR amplified using oPEB217/218. These four PCR products were used in a Gibson assembly reaction to generate a Δ*ruvB* editing plasmid. Clones were verified by Sanger sequencing with oPEB227, oPEB253, and oPEB806.

pPB180: The upstream and downstream portions of the Δ*ripX* editing template were PCR amplified using oPEB809/810 and oPEB811/812, respectively. CRISPR/Cas9 was PCR amplified using oPEB232/234 and pPB178 as the template. The pPB41 vector was PCR amplified using oPEB217/218. These four PCR products were used in a Gibson assembly reaction to generate a Δ*ripX* editing plasmid. Clones were verified by Sanger sequencing with oPEB227, oPEB253, and oPEB815.

pPB181: The ORF coding for a.a. 36-341 of YlbL was PCR amplified with primers oPEB773/677 using pPB111 as a template to incorporate the S234A mutation. The plasmid pPB12 was PCR amplified using oPEB56/57. These two PCR products were used in a Gibson assembly to generate a plasmid for overexpression of a 10xHis-Smt3-YlbL-S234A-(36-341) fusion protein in *E. coli*. Clones were verified via Sanger sequencing using oPEB527 and oPEB58.

pPB184: The upstream portion of *amyE* and the P_{xyl} promoter were PCR amplified using oPEB370/383. The chloramphenicol resistance cassette and the downstream portion of *amyE* were amplified using oPEB557/377. pPB47 was PCR amplified using oPEB116/117. The ORF of *ctpA* was PCR amplified using oPEB817/818. These four PCR products were used in a Gibson assembly reaction to generate a plasmid to integrate *ctpA* under the control of P_{xyl} at the *amyE* locus. Clones were verified by Sanger sequencing with oPEB345 and oPEB348.

pPB185: The upstream portion of *amyE* and the P_{xyl} promoter were PCR amplified using oPEB370/383. The chloramphenicol resistance cassette and the downstream portion of *amyE* were amplified using oPEB557/377. pPB47 was PCR amplified using oPEB116/117. The upstream portion of the *ctpA* ORF containing the *S297A* mutation was PCR amplified using oPEB817/820. The downstream portion of the *ctpA* ORF containing the *S297A* mutation was PCR amplified using oPEB819/818. These five PCR products were used in a Gibson assembly reaction to generate a plasmid to integrate *ctpA-S297A* under the control of P_{xyl} at the *amyE* locus. Clones were verified by Sanger sequencing with oPEB345 and oPEB348.

pPB186: The upstream portion of *amyE* and the P_{xyl} promoter were PCR amplified using oPEB370/383. The chloramphenicol resistance cassette and the downstream portion of *amyE* were amplified using oPEB557/377. pPB47 was PCR amplified using oPEB116/117. The upstream portion of the *ctpA* ORF containing the *K322A* mutation was PCR amplified using oPEB817/822. The downstream portion of the *ctpA* ORF containing the *K322A* mutation was PCR amplified using oPEB821/818. These five PCR products were used in a Gibson assembly reaction to generate a plasmid to integrate *ctpA-K322A* under the control of P_{xyl} at the *amyE* locus. Clones were verified by Sanger sequencing with oPEB345 and oPEB348.

pPB194: pDR110 was amplified using primers oPEB3F/259, resulting in the conversion of promoter P_{spac} to P_{hyp}. The *yneA* ORF was amplified using oPEB856/857. These two PCR products were used in a Gibson assembly reaction to generate a plasmid to integrate *yneA* under the control of P_{hyp} at the *amyE* locus. Clones were verified by Sanger sequencing using oPEB866 and oPEB867.

pPB200: The pET28b vector was PCR amplified using oPEB835/57. The ORF of MBP was PCR amplified using oPEB838/839 with the IDT gBlock oPEB836 as a template. The Ulp1 ORF (a.a. 403-621) was PCR amplified using oPEB840/841 with plasmid pPB13 as a template. These three PCR products were used in a Gibson assembly reaction to generate a plasmid for overexpression of the fusion protein MBP-Ulp1(403-621) in *E. coli*. Clones were verified by Sanger sequencing using oPEB527, oPEB58, and oPEB837.

pPB203: The pET28b vector was PCR amplified using oPEB835/57. The CtpA ORF (coding for a.a. 38-466) was PCR amplified using oPEB831/832. These two PCR products and the IDT gBlock oPEB836 were used in a Gibson assembly reaction to generate a plasmid for overexpression of the fusion protein MBP-Smt3-CtpA(38-466) in *E. coli*. Clones were verified by Sanger sequencing using oPEB527, oPEB58, oPEB833, and oPEB837.

pPB204: The ORF coding for a.a. 28-103 of YneA was PCR amplified using oPEB842/843. The plasmid pPB12 was PCR amplified using oPEB56/57. These two PCR products were used in a Gibson assembly to generate a plasmid for overexpression of a 10xHis-Smt3-YneA(28-103) fusion protein in *E. coli*. Clones were verified via Sanger sequencing using oPEB527 and oPEB58.

pPB214: The pET28b-MBP-Smt3 vector was PCR amplified using oPEB56/57 using pPB203 as a template. The *ctpA-S297A* ORF (coding for a.a. 38-466) was PCR amplified using oPEB831/832 using pPB185 as a template. These two PCR products were used in a Gibson assembly reaction to generate a plasmid for overexpression of the fusion protein MBP-Smt3-CtpA-S297A (38-466) in *E. coli*. Clones were verified by Sanger sequencing using oPEB527, oPEB58, and oPEB833.

pPB227: A proto-spacer targeting *fhuG* (oPEB912/913) was ligated to pPB41.

pPB228: A proto-spacer targeting *yfkH* (oPEB921/922) was ligated to pPB41.

pPB229: A proto-spacer targeting *ltaSA* (oPEB930/931) was ligated to pPB41.

pPB230: A proto-spacer targeting *ykgA* (oPEB939/940) was ligated to pPB41.

pPB231: A proto-spacer targeting *cgeE* (oPEB948/949) was ligated to pPB41.

pPB232: A proto-spacer targeting *ysnF* (oPEB957/958) was ligated to pPB41.

pPB233: A proto-spacer targeting *lytG* (oPEB966/967) was ligated to pPB41.

pPB234: A proto-spacer targeting *yxaB* (oPEB975/976) was ligated to pPB41.

pPB237: The upstream and downstream portions of the Δ*fhuG* editing template were PCR amplified using oPEB914/915 and oPEB916/917, respectively. CRISPR/Cas9 was PCR amplified using oPEB232/234 and pPB227 as the template. The pPB41 vector was PCR amplified using oPEB217/218. These four PCR products were used in a Gibson assembly reaction to generate a Δ*fhuG* editing plasmid. Clones were verified by Sanger sequencing with oPEB227, oPEB253, and oPEB920.

pPB238: The upstream and downstream portions of the Δ*yfkH* editing template were PCR amplified using oPEB923/924 and oPEB925/926, respectively. CRISPR/Cas9 was PCR amplified using oPEB232/234 and pPB228 as the template. The pPB41 vector was PCR amplified using oPEB217/218. These four PCR products were used in a Gibson assembly reaction to generate a Δ*yfkH* editing plasmid. Clones were verified by Sanger sequencing with oPEB227, oPEB253, and oPEB929.

pPB239: The upstream and downstream portions of the Δ*ltaSA* editing template were PCR amplified using oPEB932/933 and oPEB934/935, respectively. CRISPR/Cas9 was PCR amplified using oPEB232/234 and pPB229 as the template. The pPB41 vector was PCR amplified using oPEB217/218. These four PCR products were used in a Gibson assembly reaction to generate a Δ*ltaSA* editing plasmid. Clones were verified by Sanger sequencing with oPEB227, oPEB253, and oPEB938.

pPB240: The upstream and downstream portions of the Δ*ykgA* editing template were PCR amplified using oPEB941/942 and oPEB943/944, respectively. CRISPR/Cas9 was PCR amplified using oPEB232/234 and pPB230 as the template. The pPB41 vector was PCR amplified using oPEB217/218. These four PCR products were used in a Gibson assembly reaction to generate a Δ*ykgA* editing plasmid. Clones were verified by Sanger sequencing with oPEB227, oPEB253, and oPEB947.

pPB241: The upstream and downstream portions of the Δ*cgeE* editing template were PCR amplified using oPEB950/951 and oPEB952/953, respectively. CRISPR/Cas9 was PCR amplified using oPEB232/234 and pPB231 as the template. The pPB41 vector was PCR amplified using oPEB217/218. These four PCR products were used in a Gibson assembly

reaction to generate a Δ*cgeE* editing plasmid. Clones were verified by Sanger sequencing with oPEB227, oPEB253, and oPEB956.

pPB242: The upstream and downstream portions of the Δ*ysnF* editing template were PCR amplified using oPEB959/960 and oPEB961/962, respectively. CRISPR/Cas9 was PCR amplified using oPEB232/234 and pPB232 as the template. The pPB41 vector was PCR amplified using oPEB217/218. These four PCR products were used in a Gibson assembly reaction to generate a Δ*ysnF* editing plasmid. Clones were verified by Sanger sequencing with oPEB227, oPEB253, and oPEB965.

pPB243: The upstream and downstream portions of the Δ*lytG* editing template were PCR amplified using oPEB968/969 and oPEB970/972, respectively. CRISPR/Cas9 was PCR amplified using oPEB232/234 and pPB233 as the template. The pPB41 vector was PCR amplified using oPEB217/218. These four PCR products were used in a Gibson assembly reaction to generate a Δ*lytG* editing plasmid. Clones were verified by Sanger sequencing with oPEB227, oPEB253, and oPEB974.

pPB244: The upstream and downstream portions of the Δ*yxaB* editing template were PCR amplified using oPEB977/978 and oPEB979/980, respectively. CRISPR/Cas9 was PCR amplified using oPEB232/234 and pPB234 as the template. The pPB41 vector was PCR amplified using oPEB217/218. These four PCR products were used in a Gibson assembly reaction to generate a Δ*yxaB* editing plasmid. Clones were verified by Sanger sequencing with oPEB227, oPEB253, and oPEB983.

pPB267: YneA was amplified with primers oPEB1034/1035 and the plasmid pUT18C was amplified using primers oPEB1017/1018. These two PCR products were used in a Gibson

assembly reaction to generate a T18-YneA fusion for expression in BTH101 cells in a bacterial two-hybrid assay. Clones were selected on LB agar containing 100 µg/mL ampicillin and 0.2% glucose. Clones were verified via Sanger sequencing using oPEB1024 and 1025.

pPB268: YneAΔN (a.a 28-103) was amplified with primers oPEB1036/1035 and the plasmid pUT18C was amplified using primers oPEB1017/1018. These two PCR products were used in a Gibson assembly reaction to generate a T18-YneAΔN (a.a 28-103) fusion for expression in BTH101 cells in a bacterial two-hybrid assay. Clones were selected on LB agar containing 100 µg/mL ampicillin and 0.2% glucose. Clones were verified via Sanger sequencing using oPEB1024 and 1025.

pPB270: YlbL-S234A was amplified with primers oPEB1039/1040 and using pPB111 as a template. The plasmid pKT25 was amplified with primers oPEB1014/1015. These two PCR products were used in a Gibson assembly reaction to generate a T25-YlbL-S234A fusion for expression in BTH101 cells in a bacterial two-hybrid assay. Clones were selected on LB agar containing 50 µg/mL kanamycin and 0.2% glucose. Clones were verified via Sanger sequencing using oPEB1021 and 1022.

pPB271: CtpA-S297A was amplified with primers oPEB1041/1042 and using pPB185 as a template. The plasmid pKT25 was amplified with primers oPEB1014/1015. These two PCR products were used in a Gibson assembly reaction to generate a T25-CtpA-S297A fusion for expression in BTH101 cells in a bacterial two-hybrid assay. Clones were selected on LB agar containing 50 µg/mL kanamycin and 0.2% glucose. Clones were verified via Sanger sequencing using oPEB833, 1021, and 1022.

Supplemental References

1. Galinier A, Haiech J, Kilhoffer MC, Jaquinod M, Stulke J, Deutscher J, et al. The Bacillus subtilis crh gene encodes a HPr-like protein involved in carbon catabolite repression. Proceedings of the National Academy of Sciences of the United States of America. 1997;94(16):8439-44.
2. Fabret C, Hoch JA. A two-component signal transduction system essential for growth of Bacillus subtilis: implications for anti-infective therapy. Journal of bacteriology. 1998;180(23):6375-83.
3. Szurmant H, Nelson K, Kim EJ, Perego M, Hoch JA. YycH regulates the activity of the essential YycFG two-component system in Bacillus subtilis. Journal of bacteriology. 2005;187(15):5419-26.
4. Szurmant H, Mohan MA, Imus PM, Hoch JA. YycH and YycI interact to regulate the essential YycFG two-component system in Bacillus subtilis. Journal of bacteriology. 2007;189(8):3280-9.
5. Szurmant H, Bu L, Brooks CL, 3rd, Hoch JA. An essential sensor histidine kinase controlled by transmembrane helix interactions with its auxiliary proteins. Proceedings of the National Academy of Sciences of the United States of America. 2008;105(15):5891-6.
6. Fukushima T, Furihata I, Emmins R, Daniel RA, Hoch JA, Szurmant H. A role for the essential YycG sensor histidine kinase in sensing cell division. Molecular microbiology. 2011;79(2):503-22.
7. Biller SJ, Wayne KJ, Winkler ME, Burkholder WF. The putative hydrolase YycJ (WalJ) affects the coordination of cell division with DNA replication in Bacillus subtilis and may play a conserved role in cell wall metabolism. Journal of bacteriology. 2011;193(4):896-908.
8. Akerley BJ, Lampe DJ. Analysis of gene function in bacterial pathogens by GAMBIT. Methods in enzymology. 2002;358:100-8.
9. Johnson CM, Grossman AD. Identification of host genes that affect acquisition of an integrative and conjugative element in Bacillus subtilis. Molecular microbiology. 2014;93(6):1284-301.
10. Byrne RT, Chen SH, Wood EA, Cabot EL, Cox MM. Escherichia coli genes and pathways involved in surviving extreme exposure to ionizing radiation. Journal of bacteriology. 2014;196(20):3534-45.
11. van Opijnen T, Bodi KL, Camilli A. Tn-seq: high-throughput parallel sequencing for fitness and genetic interaction studies in microorganisms. Nature methods. 2009;6(10):767-72.
12. Li H, Durbin R. Fast and accurate short read alignment with Burrows-Wheeler transform. Bioinformatics (Oxford, England). 2009;25(14):1754-60.
13. Team R. RStudio: Integrated Development Environment for R: RStudio, Inc.; 2016 [Available from: http://www.rstudio.com/.
14. Benjamini Y, Hochberg Y. Controlling the false discovery rate: a practical and powerful approach to multiple testing. J R Stat Soc Ser B-Methodol. 1995;57(1):289-300.
15. Campo N, Rudner DZ. A branched pathway governing the activation of a developmental transcription factor by regulated intramembrane proteolysis. Mol Cell. 2006;23(1):25-35.
16. Burby PE, Simmons LA. MutS2 Promotes Homologous Recombination in Bacillus subtilis. Journal of bacteriology. 2017;199(2).
17. Burby PE, Simmons LA. CRISPR/Cas9 Editing of the Bacillus subtilis Genome. Bio-protocol. 2017;7(8).

18. Koo BM, Kritikos G, Farelli JD, Todor H, Tong K, Kimsey H, et al. Construction and Analysis of Two Genome-Scale Deletion Libraries for Bacillus subtilis. Cell systems. 2017;4(3):291-305.e7.
19. Gibson DG. Enzymatic assembly of overlapping DNA fragments. In: Voigt C, editor. Synthetic Biology, Pt B: Computer Aided Design and DNA Assembly. Methods in Enzymology. 498. San Diego: Elsevier Academic Press Inc; 2011. p. 349-61.
